# Evidence‐based consensus guidelines for ALS genetic testing and counseling

**DOI:** 10.1002/acn3.51895

**Published:** 2023-09-10

**Authors:** Jennifer Roggenbuck, Breda H. F. Eubank, Joshua Wright, Matthew B. Harms, Stephen J. Kolb, the ALS Genetic Testing and Counseling Guidelines Expert Panel, Senda Ajroud‐Driss, Ximena Arcila Londono, Gregory Bauer, Michael Benatar, Richard Bedlack, Benjamin Rix Brooks, Chelsea Chambers, Farid F. Chehab, Laynie Dratch, Elizabeth A. Harrington, Lauren Elman, Joseph Americo Fernandes, Laura Foster, Carlayne E. Jackson, Jamie C. Fong, Summer Gibson, Jonathan D. Glass, Stephen Goutman, Namita A. Goyal, Kelly Gwathmey, Paul Larkin, Mahesh M. Mansukhani, Weiyi Mu, Nicholas T. Olney, Erik P. Pioro, John Russo, Nadia Sethi, Carly Siskind, Jeffrey Statland, Marka M. van Blitterswijk, David Walk, Michael Weiss

**Affiliations:** ^1^ Division of Human Genetics, Department of Internal Medicine The Ohio State University Wexner Medical Center Columbus Ohio USA; ^2^ Department of Neurology The Ohio State University Wexner Medical Center Columbus Ohio USA; ^3^ Health & Physical Education Department, Faculty of Health, Community, & Education Mount Royal University 4825 Mount Royal Gate SW Calgary Alberta Canada; ^4^ Department of Neurology Columbia University Vagelos College of Physicians and Surgeons New York New York USA; ^5^ Department of Biological Chemistry & Pharmacology The Ohio State University Wexner Medical Center Columbus Ohio USA

## Abstract

**Objective:**

Advances in amyotrophic lateral sclerosis (ALS) gene discovery, ongoing gene therapy trials, and patient demand have driven increased use of ALS genetic testing. Despite this progress, the offer of genetic testing to persons with ALS is not yet “standard of care.” Our primary goal is to develop clinical ALS genetic counseling and testing guidelines to improve and standardize genetic counseling and testing practice among neurologists, genetic counselors or any provider caring for persons with ALS.

**Methods:**

Core clinical questions were identified and a rapid review performed according to Preferred Reporting Items for Systematic Reviews and Meta‐Analyses (PRISMA‐P) 2015 method. Guideline recommendations were drafted and the strength of evidence for each recommendation was assessed by combining two systems: the Grading of Recommendations, Assessment, Development and Evaluation (GRADE) System and the Evaluation of Genomic Applications in Practice and Prevention (EGAPP). A modified Delphi approach was used to reach consensus among a group of content experts for each guideline statement.

**Results:**

A total of 35 guideline statements were developed. In summary, all persons with ALS should be offered single‐step genetic testing, consisting of a *C9orf72* assay, along with sequencing of *SOD1, FUS,* and *TARDBP,* at a minimum. The key education and genetic risk assessments that should be provided before and after testing are delineated. Specific guidance regarding testing methods and reporting for *C9orf72* and other genes is provided for commercial laboratories.

**Interpretation:**

These evidence‐based, consensus guidelines will support all stakeholders in the ALS community in navigating benefits and challenges of genetic testing.

## Background

Rapid progress in the discovery of amyotrophic lateral sclerosis (ALS)‐associated genes, and a growing recognition of the genetic basis of clinically sporadic ALS, has opened the door to an era of gene‐targeted therapies for persons with ALS. Despite the progress in ALS gene discovery, and a wide array of clinical gene testing options, the offer of genetic testing to people with ALS is not yet broadly considered “standard of care” and many people with ALS who desire access to genetic testing are not offered it.

A growing proportion of clinicians offer genetic testing to persons with familial ALS, though only 10–50% of clinicians offer testing in the case of apparently sporadic ALS.^1^, ^2^, ^3^, ^4^, ^5^ Persons with ALS value the utility of genetic testing as part of ALS clinical management, regardless of the presence or absence of family history.^6^, ^7^ Surveys of those who have had ALS genetic testing have identified a need for more complete genetic counseling and risk assessment, such as information pertaining to the implications of test results for relatives.^6^, ^8^, ^9^ In addition to the problem of inconsistent genetic testing and counseling practices, concerns have been raised regarding testing laboratory methodologies, particularly with respect the detection and reporting of the *C9orf72* expansion.^10^, ^11^, ^12^

Despite these challenges, recently reaffirmed US care guidelines do not address the offer of genetic testing^13^ (reaffirmed February 25, 2023), and European guidelines specify that ALS genetic testing should be offered only to patients with familial ALS or the *SOD1* D90A phenotype.^14^ The development of evidence‐based, consensus guidelines will provide clinicians with a framework for the offer of genetic testing and outline the information that should be provided to patients before and after testing. In addition, these guidelines will provide specific recommendations regarding test methods and reporting, thus providing guidance to both clinicians and testing laboratories in navigating the challenges of this technology and supporting equitable patient access to genetic diagnosis and gene‐targeted therapies.

The first gene‐targeted treatment for ALS, an antisense oligonucleotide therapy for *SOD1* ALS, was recently granted accelerated approval by the FDA.^15^ The need for consistent genetic testing practices and patient access to testing and counseling is particularly acute as gene‐targeted clinical trials are ongoing and in development for many other ALS‐associated genes.^16^ Identification of relatively rare genetic forms of ALS is dependent upon widespread genetic characterization of patient populations, which has been successfully applied in large scale research and clinical testing efforts,^17^, ^18^ but yet to be incorporated into routine clinical practice nationwide.

## Methods

### Core clinical questions

We identified core clinical questions to be addressed by the guidelines using the AGREE II instrument, an international tool routinely employed in medical guideline development and evaluation by professional organizations. The AGREE II (Appraisal of Guidelines, Research and Evaluation) instrument is an international system created to advance guideline development in healthcare.^19^ The AGREE II reporting checklist was created in 2016 to “assist guideline developers to improve the completeness and transparency of reporting in their practice guidelines”.^19^ This checklist provides guideline authors or reviewers with a step‐by‐step structure to develop and/or evaluate a high‐quality practice guideline. The checklist reflects the AGREE II's structure of six quality domains and 23 key items, providing a systematic and logical process for reporting essential information. Since its publication in 2010, the AGREE II instrument has been applied and/or cited in over 900 publications creating or evaluating practice guidelines.

Each of the core clinical questions we identified covers a content domain relevant for the development of ALS genetic testing guidelines: a clinical testing domain, a genetic counseling domain, and a laboratory methods domain. These questions formed the foundation for the rapid review (defined below):

1. What genetic testing should be offered to persons with ALS? (clinical testing domain)
2. What information should be provided to persons with ALS before and after testing? (genetic counseling domain)
3. What test methodologies, reporting, and interpretation standards should be used? (laboratory methods domain)

### Clinical scope

The target patient population to which the guidelines are intended to apply are persons diagnosed with ALS. That stated, we recognize that in clinical genetics, the family is often considered the unit of care, and indeed family members of a person with ALS may directly or indirectly receive care or education from the proband's neurologist, genetic counselor, or other clinician.

### Author and expert groups

The author group includes a genetic counselor (JR), physician scientist (SK), and clinician researcher (MH), all with expertise and published research in ALS genetics, genetic counseling, testing approaches, outcomes, and laboratory methods, as well as a guidelines methodologist (BE), with expertise in medical guideline development using the modified Delphi approach. Expert Panel participants were purposely recruited to represent each stakeholder group and a range of disciplines, expertise, and geographic representation across the United States. Experts were chosen to represent academic neurologists, community neurologists, genetic counselors, physician scientists, laboratory experts, ALS advocates, and persons with ALS. Persons with ALS were included to represent the patient perspective, which is increasingly recognized as key to effective care.^20^, ^21^, ^22^, ^23^

### Rapid review

A rapid review of the literature was performed according to Preferred Reporting Items for Systematic Reviews and Meta‐Analyses (PRISMA‐P) 2015 guidelines. Three electronic databases (Medline, EMBASE, and CINAHL) were searched from inception to March 2022. Search terms included any of genetic predisposition to disease, genetic counseling, genetic testing, genetic variation, genes, mutation, missense, DNA analysis, mutational analysis, mutation rate, penetrance, sequence analysis, pedigree; any of *C9ORF72, FUS, OPTN, TBK1, KIF5A, VCP, ANXA11, MATR3, SQSTM1, ANG, hnRNPA2B1, hnRNPA1, CHCHD10, DCTN1, PRPH, TAF15, TIA1* (or written out version of gene names, synonyms), and “Amyotrophic lateral sclerosis” or “ALS”. The search strategy is presented in the Evidence Summary Document, available in Supplementary Materials.

**Figure 1:**
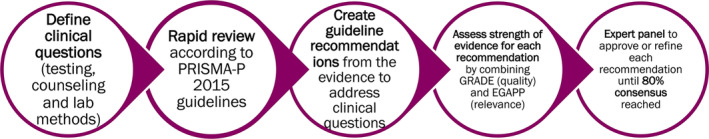
Summary of methods for guidelines development. GRADE; Grading of Recommendations, Assessment, Development and Evaluation^19^; EGAPP, Evaluation of Genomic Applications in Practice and Prevention.^24^

**Figure 2:**
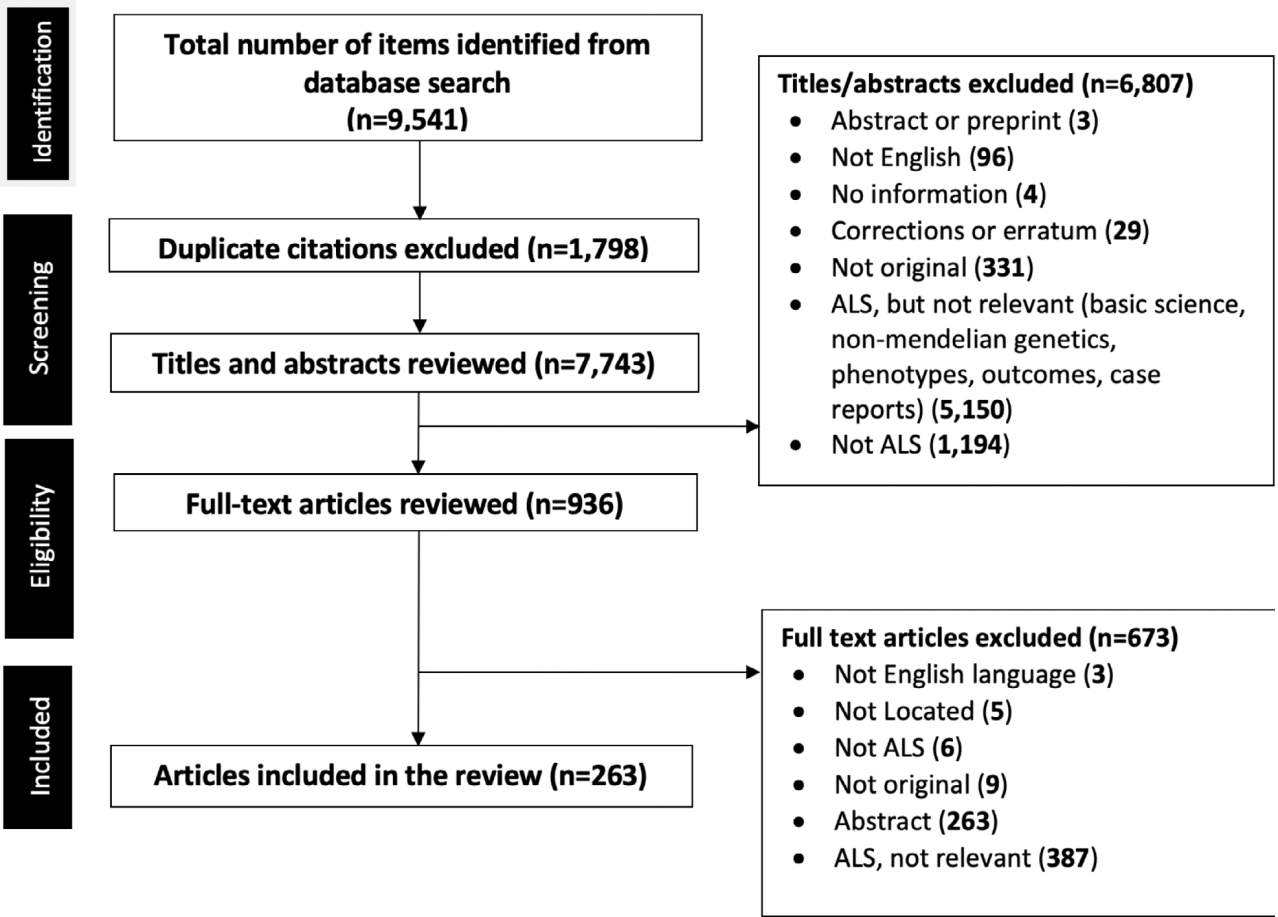
PRISMA‐P flow diagram of the identified studies (263 articles were included in the rapid review).

A total of 9541 potentially relevant articles were identified in the initial search. After 1794 duplicates were removed, 7743 citations underwent title and abstract review by JR, MH, and SK. After applying inclusion and exclusion criteria to the title and corresponding abstracts and resolving any nonunanimous decisions, 936 were selected for full‐text review. After a full‐text article review, 263 articles were selected for inclusion, and key data extracted and summarized (Figs. 1 and 2).

### Initial ALS guideline development and the modified Delphi consensus

The evidence retrieved from the rapid review was summarized, used to draft initial guideline recommendations, and mapped to each domain: 7 recommendations concerning clinical genetic testing; 19 recommendations concerning genetic counseling; and 9 recommendations concerning laboratory methods and reporting. See Evidence Summary Document, Table S1. Each recommendation was evaluated for strength of evidence by adapting two systems: (1) the Grading of Recommendations, Assessment, Development and Evaluation (GRADE) System,^19^ and (2) the Evaluation of Genomic Applications in Practice and Prevention (EGAPP).^24^ GRADE is a method for assessing the quality of evidence when developing health care guidelines and determining whether an intervention is justified. It is considered the gold standard for assessing evidence in medicine and relies on randomized control trials (RCTs) for weighing quality; wherein recommendations are assigned a grade of high (A), moderate (B), low (C), or very low (D) based on the strength of the supporting evidence. Given the paucity and impracticality of RCTs in genetic studies, we adapted the EGAPP criteria for determining quality and relevance to our evidence base. Each included paper was mapped to one of three evidence domains (clinical validity, analytical validity, and clinical utility) and scored for quality based on study attributes such as cohort size, case–control matching, rigor of variant interpretation, and other features. See Table 1.

**Table 1:** Methodological quality of individual studies (EGAPP) grading.

Level	Clinical validity (variant frequency studies)	Analytical validity (laboratory studies)	Clinical utility (Pt outcome studies, genetic risk assessment)
I	American College of Medical Genetics (ACMG) variant criteria used, large case–control study, population register of >500 or more Well‐designed longitudinal studies, validated clinical decision rule Cases: >500 consecutive or population, controls: >1000, reasonably matched Variant calling: stringent or explicit Meta‐analysis with homogeneity	Blinded lab method studies, validation of methods to controls Collaborative study using a large panel of well characterized samples	Single randomized controlled trial (RCT) Meta‐analysis of RCT
II	ACMG criteria used, large case–control study, population register of <500 or more Well‐designed case–control studies Cases: 100–500 consecutive/pop or >500 convenience Controls: 500–1000, reasonably matched Variant calling: stringent or explicit Meta‐analysis with heterogeneity	Lab method studies, validation of methods, but not to controls Comparison or reporting of lab methods Other data from proficiency testing schemes Well‐designed peer‐reviewed studies (method comparisons, validation studies) Expert Panel FDA studies	Case–control studies Twin studies Meta‐analysis with heterogeneity
III	ACMG criteria not used or not case–control Lower quality case–control or cross‐sectional, unvalidated clinical decision rule Cases: 100–500 convenience Controls: 100–500, less well‐matched Variant calling stringent or explicit		Cohort or case–control Controlled trial without randomization Patient surveys Practice surveys
IV	Case series, unpublished and non‐peer reviewed research, consensus guidelines Cases: <100, controls: <100 or no controls regardless of size Variant calling: not stringent or explicit	Clinician practice surveys Unpublished and/or non‐peer reviewed research Studies on performance of the same basic methodology, but used to test for a different target	Case‐series Unpublished and non‐peer reviewed studies Clinic lab or manufacturer data Consensus guidelines
V	Expert opinion	Expert opinion	Expert opinion

Adapted from Teutsch et al.^24^

A three‐round modified Delphi approach was used to revise and finalize recommendations. The modified Delphi approach has been used in medical settings to achieve consensus for a defined clinical problem.^25^, ^26^, ^27^, ^28^, ^29^, ^30^, ^31^, ^32^, ^33^, ^34^, ^35^ This iterative method utilizes repeated rounds of voting to progress systematically toward question resolution. Draft recommendations were circulated to an expert group, which was asked to vote “yes” or “no” on each statement. Respondents were given the option to abstain from voting on statements by selecting “insufficient knowledge to assess.” Prior to voting, consensus was defined as ≥80% of experts voting in agreement or against a statement (i.e., the summative of “yes” or “no”). This level of consensus has been advocated to achieve content validity when there are at least 10 experts participating in consensus development.^26^, ^36^

## Results

**Figure 3:**
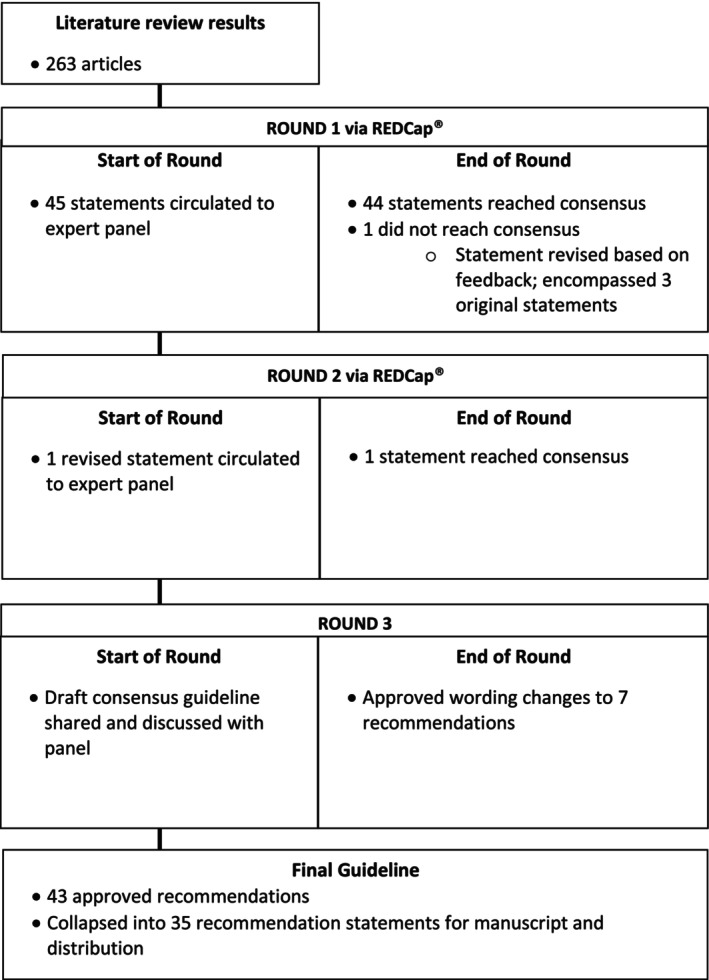
Modified Delphi methodology and results.

The first two rounds of voting were completed via an email link to a Research Electronic Data Capture (REDCap®) survey hosted at the Ohio State University.^37^, ^38^ One recommendation statement not reaching consensus after Round 1 (“*All persons with ALS of European descent should be offered C9orf72 testing as the first genetic test*”) was revised according to written feedback provided by the expert group. The revised recommendation, “*All persons with ALS should be offered testing with an ALS gene panel that includes C9orf72*” reached 100% consensus. The final round (Round 3) consisted of a face‐to‐face meeting to discuss and vote on minor changes to the wording of 7 recommendation statements. See Figure 3. The Round 3 meeting was held on October 17, 2022 via the web‐based platform Zoom Video Communications™, version 5.10.0.

**Figure 4:**
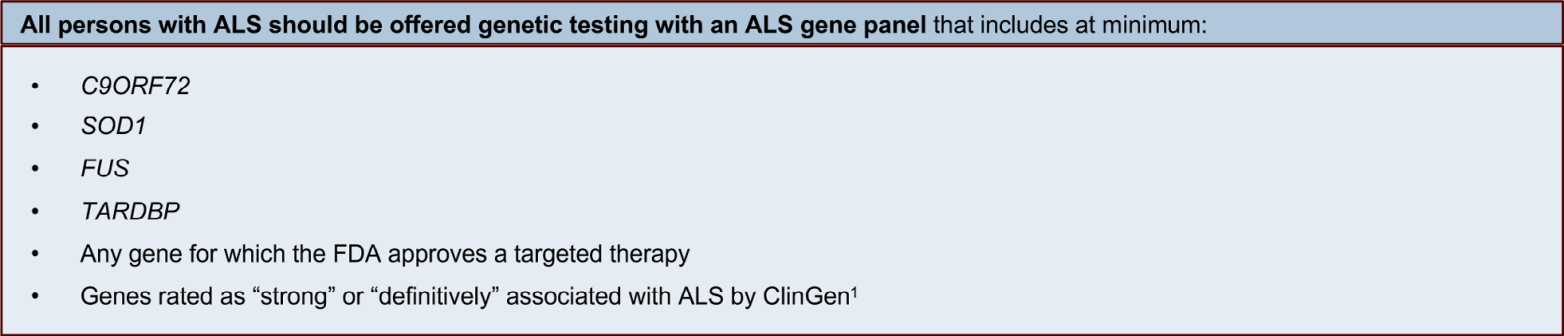
Summary of clinical testing guidelines. ClinGen, Clinical Genome Resource, https://clinicalgenome.org/affiliation/40096/.

**Figure 5:**
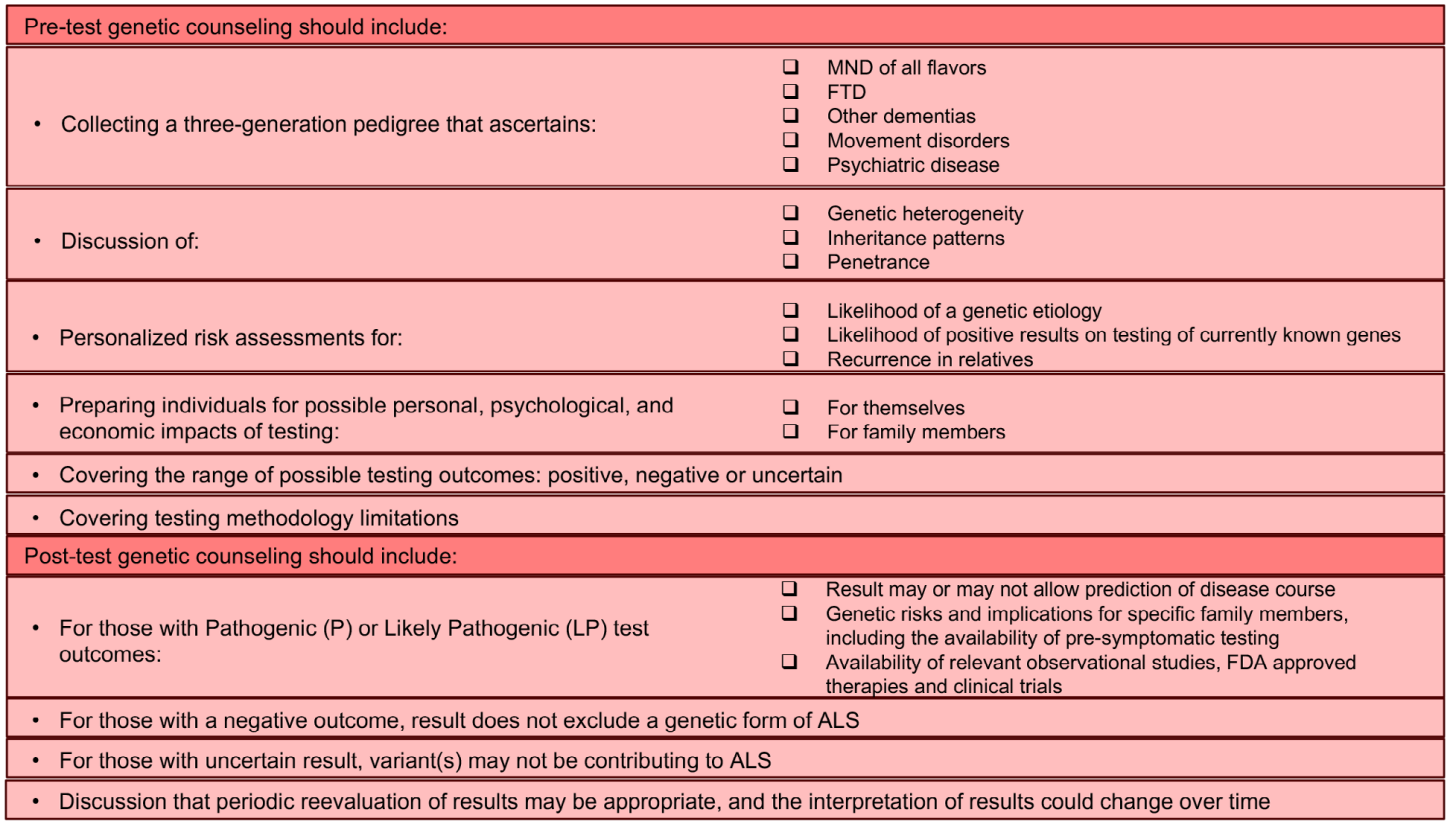
Summary of genetic counseling recommendations.

**Figure 6:**
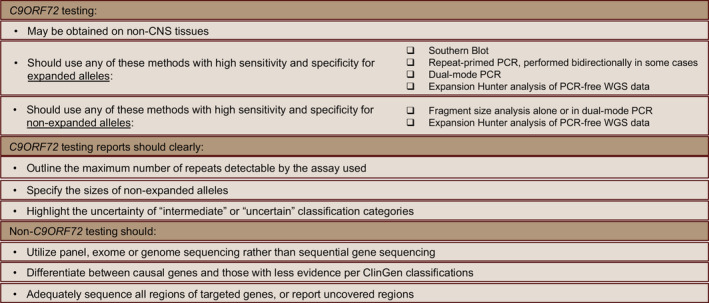
Summary of laboratory recommendations. PCR, polymerase chain reaction; WGS, whole‐genome sequencing.

At the conclusion of the modified Delphi Process, 43 recommendation statements were finalized and approved, forming our Guidelines. After related recommendation statements were merged for brevity, 35 recommendations remained, encompassing the domains of clinical testing (7 recommendations, summarized in Fig. 4), genetic counseling (19 recommendations, summarized in Fig. 5), and laboratory methods and reporting (9 recommendations, summarized in Fig. 6). Each Guideline below reflects a component of our recommended practice for genetic testing and counseling in ALS. Key points summarizing the evidence and/or clinical context are provided after each Guideline, followed by the GRADE rating (quality of evidence) and corresponding Strength of the recommendation.

### Guidelines for the offer of clinical genetic testing to persons with ALS

**Recommendation 1: All persons with ALS should be offered genetic testing.**

*Key points*: In populations of European geoancestry, pathogenic or likely pathogenic variants may be found in the majority of familial ALS cases and in a minority of apparently sporadic cases.^39^, ^40^, ^41^ The frequencies of known ALS variants appear lower in other studied populations, though more data are needed.^42^ Persons with ALS desire access to genetic testing and perceive benefits from it, irrespective of family history status or test outcome.^6^, ^7^ Because the yield of genetic testing is sufficiently high and will lead to therapeutic intervention in some, the offer of testing should be universal.

GRADE rating: A ‐ Strong.

Strength: This recommendation is supported by at least one study of Level 1 evidence.

**Recommendation 2: All persons with ALS should be offered testing with an ALS gene panel that includes *C9orf72*.**

*Key points*: The *C9orf72* repeat expansion demonstrates incomplete penetrance and is the most common genetic cause of ALS in European‐ancestry populations, accounting for 1 in 10 cases, irrespective of the presence or absence of a family history of ALS or FTD.^39^, ^43^, ^44^, ^45^, ^46^, ^47^ The expansion has also been identified at lower frequencies in other populations.^48^, ^49^, ^50^, ^51^, ^52^ This frequency justifies the offer of *C9orf72* testing to all persons with ALS.

GRADE rating: A ‐ Strong.

Strength: This recommendation is supported by at least one study of Level 1 evidence.

**Recommendation 3: All persons with ALS should be offered testing with an ALS gene panel that includes *SOD1*.**

*Key points*: Pathogenic variants in *SOD1* have been identified in multiple ALS cohorts around the world, and may represent the leading genetic cause of ALS in many populations.^43^, ^44^, ^48^, ^49^ The recent FDA approval of tofersen, an antisense oligonucleotide therapy to *SOD1*, underscores the importance of universal access to *SOD1* testing.^15^

GRADE rating: A ‐ Strong.

Strength: This recommendation is supported by at least one study of Level 1 evidence.

**Recommendation 4: All persons with ALS should be offered testing with an ALS gene panel that includes *FUS*.**

*Key points*: Pathogenic variants in FUS have been identified in multiple ALS cohorts around the world, and have been reported to occur de novo in juvenile ALS.^43^, ^44^, ^48^, ^49^ Identification of a pathogenic variant in FUS may lead to eligibility for ongoing clinical trials.

GRADE rating: A ‐ Strong.

Strength: This recommendation is supported by at least one study of Level 1 evidence.

**Recommendation 5: All persons with ALS should be offered testing with an ALS gene panel that includes *TARDBP*.**

*Key points*: Pathogenic variants in TARDBP have been identified in multiple ALS cohorts around the world^43^, ^44^, ^48^, ^49^ at a frequency around 1% which warrants the offer of testing.

GRADE rating: A ‐ Strong.

Strength: This recommendation is supported by at least one study of Level 1 evidence.

**Recommendation 6: Additional genetic testing should include genes strongly and definitively associated with ALS as determined by ClinGen.**

*Key points*: Many reported ALS genes lack sufficient evidence for causality. The Amyotrophic Lateral Sclerosis Spectrum Disorders Gene Curation Expert Panel of ClinGen (https://clinicalgenome.org/affiliation/40096/) follows a standardized approach for reviewing genetic and experimental evidence in assigning gene‐disease validity in ALS. Genes classified with “definitive” disease validity are appropriate to include in clinical testing.

GRADE rating: D ‐ Expert.

Strength: This recommendation is supported by Expert Opinion.

**Recommendation 7: In the event of an FDA‐approved gene‐targeted therapy, all persons with ALS should be offered testing for that gene.**

*Key points*: The opportunity for FDA‐approved gene‐targeted treatment warrants the offer of genetic testing for the corresponding gene. The first gene‐targeted therapy for ALS was recently granted accelerated approval by the FDA.

GRADE rating: D ‐ Expert.

Strength: This recommendation is supported by Expert Opinion.

### Guidelines for genetic counseling in persons with ALS

**Recommendation 8: Genetic counseling and education should be provided to all persons with ALS.**

*Key points*: Genetic counseling promotes adaptation to the occurrence or risk for disease that may be hereditary, including the nature of inheritance, understanding of genetic testing options, and implications for family members. All persons with ALS should be offered genetic counseling, irrespective of the presence or absence of a family history of ALS.^42^, ^53^, ^54^, ^55^ Sporadic ALS demonstrates high heritability in twin studies^56^ and pathogenic genetic variants may be identified in at least 10%.^42^ While ideally provided by a board‐certified genetic counselor, other health professionals, such as neurologists and nurse practitioners, may provide this counseling when genetic counselors are not available.

GRADE rating: C ‐ Weak.

Strength: This recommendation is supported by at least one study of Level 3 evidence.

**Recommendation 9. Genetic counseling should precede the offer of testing.**

*Key points*: Genetic counseling should be provided before testing, to empower persons with ALS to weigh the potential benefits, risks and limitations of testing, and anticipate the possible impact of testing on themselves and their family members.^54^, ^55^, ^57^ A variety of potential harms to both affected persons and family members can arise from the use of genetic testing without pretest counseling, including those related to uncertainties around incomplete penetrance, limitations of laboratory methods, variants of unknown significance, and genetic discrimination as well as legal, social, and psychological sequalae.^8^, ^42^

GRADE rating: C ‐ Weak.

Strength: This recommendation is supported by at least one study of Level 3 evidence.

**Recommendation 10. A pedigree going back three generations at minimum should be documented.**

*Summary of evidence*: Although increasing the number of individuals ascertained in family history also increases the likelihood that affected persons will be identified by chance,^58^ the penetrance of many ALS pathogenic variants is incomplete and pedigree documentation is a fundamental component of ALS genetic risk assessment and genetic counseling.^42^, ^53^, ^54^, ^55^ Pedigree data will also inform genetic risk assessment prior to genetic testing or when genetic testing is negative or inconclusive.^55^

GRADE rating: B ‐ Moderate.

Key points: This recommendation is supported by at least one study of Level 2 evidence.

**Recommendation 11. The pedigree should ascertain ALS and related motor neuron disorders (e.g., primary lateral sclerosis, progressive muscular atrophy, pseudobulbar atrophy), frontotemporal dementia, other dementias, movement disorders, and psychiatric disease.**

*Key points*: Studies of clinic‐based ALS cohorts and defined patient populations have consistently shown that the incidence of pathogenic ALS variants is higher in probands who have a family history of ALS.^40^, ^41^, ^58^, ^59^ However, other neurodegenerative phenotypes may share a common genetic etiology with ALS, most notably frontotemporal dementia (FTD), as well as primary lateral sclerosis, progressive muscular atrophy, pseudobulbar palsy, parkinsonism, and psychiatric disease.^53^, ^60^, ^61^, ^62^ Additionally, given the challenge of discerning dementia types, the presence of unspecified or other dementia types should also be documented in the pedigree.^40^, ^59^, ^63^

GRADE rating: B ‐ Moderate.

Strength: This recommendation is supported by at least one study of Level 2 evidence.

**Recommendation 12: Genetic counseling should include personalized risk assessments for the likelihood of a genetic etiology, and the likelihood of positive results on testing of currently known genes**.

*Key points*: The likelihood of a genetic etiology in a person or family with ALS varies with clinical features such as family history, age of onset of symptoms, and the presence of additional phenotypes such as FTD.^58^, ^64^ For those who have one or more first‐ or second‐degree relatives affected with ALS or FTD, the possibility of dominant transmission should be considered and discussed.^55^, ^65^ The likelihood of a positive result with genetic testing is higher in those with close and/or multiple affected relatives.^65^, ^66^ For those with no known family history, earlier onset of symptoms indicates a somewhat higher likelihood of an identifiable genetic etiology,^40^, ^67^ as does concomitant FTD.^64^

GRADE rating: B ‐ Moderate.

Strength: This recommendation is supported with at least one study of Level 2 evidence.

**Recommendation 13: Genetic counseling should include discussion of genetic heterogeneity.**

*Key points*: Pathogenic variants in growing number of genes have been shown to cause or increase the risk for ALS; variant frequencies differ by geoancestry.^58^, ^68^, ^69^ Education regarding the genetic heterogeneity of ALS prepares probands and their families for the uncertainty that may arise in risk assessment and genetic testing.^42^, ^70^

GRADE rating: B ‐ Moderate.

Strength: This recommendation is supported by at least one study of Level 2 evidence.

**Recommendation 14: Genetic counseling should include a discussion of inheritance patterns.**

*Key points*: Although ALS variants can be transmitted in an autosomal dominant, autosomal recessive or X‐linked manner,^71^ the overwhelming majority are dominant. Possible or suspected inheritance patterns should be discussed with persons with ALS, even in the absence of a positive family history.^53^, ^55^ Persons with a genetic form of ALS may or may not have a recognized family history of affected relatives and may transmit the variant and associated disease risk to children.^42^, ^58^

GRADE rating: B ‐ Moderate.

Strength of recommendation: This recommendation is supported with at least one study of Level 2 evidence.

**Recommendation 15: Genetic counseling should include a discussion of penetrance.**

*Key points*: Many ALS variants are incompletely penetrant, meaning that not all carriers of the variant will develop disease. Penetrance studies have estimated a high disease risk for some *SOD1* (e.g., A5V) and *FUS* variants, but much lower disease risk for others, including the *C9orf72* expansion.^42^, ^47^, ^72^, ^73^, ^74^, ^75^, ^76^, ^77^ Genetic counseling should convey the variable and uncertain penetrance of most ALS variants, helping persons with ALS appreciate the limitations of genetic testing in predicting disease in family members.^5^, ^8^, ^53^, ^55^, ^78^

GRADE rating: C ‐ Weak.

Strength of recommendation: This recommendation is supported with at least one study of Level 3 evidence.

**Recommendation 16: Genetic counseling should include personalized risk assessment for recurrence in relatives**.

*Key points*: For persons with ALS who have no known family history of ALS or FTD, empiric data may be used to estimate the risk that relatives would develop ALS. In European populations, the lifetime risk of ALS in first‐degree relatives of those with apparently sporadic disease appears to be 1–3%.^79^, ^80^, ^81^ For those who have a first‐ or second‐degree relative affected with ALS or FTD, genetic risk assessment for family members should be informed by pedigree analysis.^55^ Discussion of personalized risks based on family history serves to introduce and contextualize the implications of genetic risk.^42^

GRADE rating: B ‐ Moderate.

Strength of recommendation: This recommendation is supported with at least one study of Level 2 evidence.

**Recommendation 17: Pretest counseling should prepare individuals for possible personal, psychological, and economic impacts of testing on themselves and their family members.**

*Key points*: Pretest communication of the potential psychosocial impact of testing helps persons with ALS anticipate possible outcomes and prepare for uncertainty. Individual motivations for testing should be discussed.^9^, ^53^, ^57^ Studies have shown that persons with ALS weigh the implications for relatives in their testing decision; family communication and support should be explored in pretest counseling.^9^ Persons with ALS should be encouraged to reflect on whether results will be shared with family members and anticipate the ways that various test outcomes may impact their family with respect to genetic risk, genetic privacy, and discrimination.^9^, ^53^, ^57^, ^78^, ^82^

GRADE rating: C ‐ Weak.

Strength: This recommendation is supported with at least one study of Level 3 evidence.

**Recommendation 18: Persons with ALS should be informed of the range of possible testing outcomes: positive, negative, or uncertain.**

*Key points*: Outcomes for clinical ALS genetic testing may include positive, negative, uncertain, and/or indeterminate result interpretations.^11^, ^40^, ^41^ All potential outcomes should be discussed prior to testing, with particular care to prepare individuals for the challenge of an uncertain result.^8^, ^9^, ^55^, ^70^

GRADE rating: C ‐ Weak.

Strength of recommendation: This recommendation is supported with at least one study of Level 3 evidence.

**Recommendation 19: Persons with ALS and their families should be informed that all testing methodologies have limitations.**

*Key points*: Technical limitations in ALS genetic testing may produce false‐negative or false‐positive results. Persons considering ALS genetic testing should understand that current technologies may in some cases fail.^5^, ^8^, ^9^, ^42^, ^57^, ^70^, ^72^, ^83^ Assays to detect the *C9orf72* expansion may fail to identify expanded allele. There is no validated cutoff that differentiates between pathogenic and nonpathogenic alleles, and the clinical significance of intermediate size alleles is unknown. Multigene sequencing panels may fail to detect, erroneously identify, or misinterpret a genetic variant for a variety of reasons. Recommendations for assay use and reporting to address these issues can be found below.

GRADE rating: C ‐ Weak.

Strength: This recommendation is supported with at least one study of Level 3 evidence.

**Recommendation 20: All persons with ALS who have genetic testing should receive posttest counseling**.

*Key points*: Posttest counseling provides persons with ALS the opportunity to discuss their result and understand the implications in the context of their specific personal and family circumstances.^6^, ^7^, ^9^, ^54^, ^55^, ^57^, ^84^

GRADE rating: C ‐ Weak.

Strength: This recommendation is supported with at least one study of Level 3 evidence.

**Recommendation 21: Posttest counseling should inform persons with ALS with a pathogenic or likely pathogenic test outcome that result may or may not allow prediction of disease course.**

*Key points*: Genotype–phenotype correlations in ALS are generally limited to trends in grouped data and have low predictive value in individual cases, with some notable exceptions. When a pathogenic or likely pathogenic variant is identified, persons with ALS should understand that the genetic result does not allow prediction of disease course in most cases.^5^, ^8^, ^42^, ^53^, ^54^, ^55^, ^57^, ^72^, ^75^, ^76^, ^82^, ^83^, ^85^

GRADE rating: B ‐ Moderate.

Strength of recommendation: This recommendation is supported with at least one study of Level 2 evidence.

**Recommendation 22. Posttest counseling should inform persons with ALS with a pathogenic or likely pathogenic test outcome of the genetic risks and implications for specific family members, including the availability of presymptomatic testing.**

*Key points*: When a pathogenic or likely pathogenic variant is identified in a person with ALS, the family history should be reviewed in the context of the likely inheritance pattern of the variant, and implications and genetic risks for close and extended family members should be discussed. Adult relatives are candidates for presymptomatic testing, which is a personal choice and should be performed with appropriate genetic counseling.^6^, ^9^, ^55^, ^57^, ^71^, ^82^, ^83^

GRADE rating: C ‐ Weak.

Strength of recommendation: This recommendation is supported with at least one study of Level 3 evidence.

**Recommendation 23. Posttest counseling should inform persons with ALS with a pathogenic or likely pathogenic test outcome of the availability of relevant observational studies, FDA‐approved therapies, and clinical trials.**

*Key points*: Persons with ALS identified to have a pathogenic or likely pathogenic variant should be informed of FDA‐approved or investigational therapies which are targeted to their particular gene; opportunities for gene‐targeted interventions are likely to increase in coming years.^41^, ^42^, ^84^ Likewise, the availability of observational studies (gene‐specific and otherwise) should be reviewed.^57^, ^70^ Clinical trial and other research opportunities can be identified via clinicaltrials.gov and other resources.

GRADE rating: C ‐ Weak.

Strength: This recommendation is supported with at least one study of Level 3 evidence.

**Recommendation 24. Posttest counseling should inform persons with ALS with a negative outcome that the result does not exclude a genetic form of ALS.**

*Key points*: Current genetic testing fails to identify a genetic etiology in a significant proportion of familial cases, indicating that additional, unknown genetic mechanisms contribute to the etiology of ALS. First‐degree relatives of individuals with familial or sporadic ALS without an identifiable genetic basis remain at increased risk of developing ALS compared with the general population.^86^ Posttest counseling should convey and help families adapt to the uncertainty that may remain after clinical genetic testing, particularly for those with a positive family history. DNA banking or referral to gene‐discovery studies may be offered in such cases.

GRADE rating: D ‐ Expert.

Strength: This recommendation is supported with at least one study of Level 5 evidence and expert opinion.

**Recommendation 25. Posttest counseling should inform persons with ALS with an uncertain result that the variant(s) may or may not be contributing to their ALS.**

*Key points*: A significant proportion of patients who undergo ALS genetic testing will receive an uncertain result.^12^, ^40^, ^41^ Many variants of uncertain significance are likely to be incidental, and avenues for further investigation may be limited. In such cases, posttest counseling should emphasize the unknown clinical significance of the result, acknowledging the potential harms of misinterpreting a variant.^5^, ^18^, ^40^, ^42^, ^55^

GRADE rating: C ‐ Weak.

Strength: This recommendation is supported with at least one study of Level 3 evidence.

**Recommendation 26. Posttest counseling should inform persons with ALS that periodic reevaluation of genetic results may be appropriate, and that the interpretation of their results could change over time.**

*Key points*: As new evidence emerges, commercial laboratories and/or clinical teams may change their interpretation of specific genetic variants, and persons with ALS should be informed that reinterpretation of clinical testing may occur.^42^ Additional genetic testing may be appropriate as new ALS‐associated genes are identified.

GRADE rating: D ‐ Expert.

Strength: This recommendation is supported with at least one study of Level 5 evidence and expert opinion.

### Guidelines for laboratory methods and reporting

**Recommendation 27. Testing performed on DNA derived from non‐CNS tissues is sufficient to establish the presence of a *C9orf72* repeat expansion.**

*Key points*: Although the *C9orf72* expansion shows extreme somatic instability and produces different sized repeats longitudinally and across tissues in one individual, there are no documented cases where this variability produced normal testing in peripheral tissues despite an expanded repeat lengths in the central nervous system. DNA derived from blood or other non‐CNS tissues can therefore be used to test for the presence or absence of *C9orf72* repeat expansions.^83^, ^87^, ^88^

GRADE rating: B ‐ Moderate.

Strength: This recommendation is supported by at least one study of Level 2 evidence.

**Recommendation 28. *C9orf72* testing should use a method with high sensitivity and specificity for expanded alleles.**

*Key points*: A variety of assays are used in commercial laboratories to detect *C9orf72* expansions. In a 2014 blinded study, only 5 out of 14 laboratories reported PCR‐based *C9orf72* results in complete concordance with the reference Southern blot result, and both false‐negative and false‐positive results were identified.^88^ Deficiencies were correlated with the type of assay being used, necessitating use and specification of appropriate assays in these recommendations.

GRADE rating: A ‐ Strong.

Strength: This recommendation is supported by one study of Level 1 evidence.

**Recommendation 28a. Southern blot is an acceptable method for detecting expanded *C9orf72* alleles with high sensitivity and specificity.**

*Key points*: Southern blot analysis is considered the gold standard for detecting the expansion and is the only commercial method currently available for sizing large expansions. Southern blot may fail to distinguish intermediate or smaller expansions from normal alleles^10^, ^89^ and will need to be supplemented with a method capable of accurately sizing these alleles to meet Recommendation 29.

GRADE rating: A ‐ Strong.

Strength: This recommendation is supported by one study of Level 1 evidence.

**Recommendation 28b. Repeat‐primed PCR, performed bidirectionally in some circumstances, is an acceptable method for detecting expanded *C9orf72* alleles with high sensitivity and specificity.**

*Key points*: RP‐PCR assays are cost‐effective, rapid, and identify patients with an expanded allele by revealing a “saw‐tooth pattern” when a significant repeat expansion is present. However, sequence variants adjacent to the expansion may result in deviant RP‐PCR curves leading to false‐negatives. In such cases, the use of two RP‐PCR assays, one from either end of the repeat region will resolve both alleles with appropriate sensitivity and specificity.^10^, ^90^, ^91^

GRADE rating: A ‐ Strong.

Strength: This recommendation is supported by one study of Level 1 evidence.

**Recommendation 28c. Dual‐mode PCR is an acceptable method for detecting expanded *C9orf72* alleles with high sensitivity and specificity.**

*Key points*: The dual‐mode long read PCR assay described by Bram et al. is capable of amplifying GC‐rich sequence and enables repeat sizing from 2 to ∼950 repeats, detects expansions of >950 repeats in agreement with other assays, and flags sequence variants around the repeat tract.^92^

GRADE rating: B ‐ Moderate.

Strength: This recommendation is supported by one study of Level 2 evidence.

**Recommendation 28d. Expansion Hunter analysis of PCR‐free whole‐genome sequencing data is an acceptable method for detecting expanded *C9orf72* alleles with high sensitivity and specificity.**

*Key points*: The Expansion Hunter Software Tool developed and validated by Dolzhenko et al. can identify the presence or absence of the *C9orf72* expansion when used on PCR‐free WGS short‐read data, even if the expanded repeat is longer than the read length.^93^ Although sensitivity for the presence or absence of the repeat is high, the size prediction is an estimate and cannot be taken as the true size. Laboratories may want to validate predicted expansions with an orthogonal method.

GRADE rating: B ‐ Moderate.

Strength: This recommendation is supported by one study of Level 2 evidence.

**Recommendation 29. *C9orf72* testing should use a method that accurately sizes normal range alleles.**

*Key points*: A method that allows determination of the exact repeat numbers of alleles with up to 30 repeats is able to exclude a pathological repeat expansion if two different alleles in the wild‐type range are detected.^10^ Having normal alleles sized and reported clearly on laboratory reports will enable clinicians to assess the risk of a false‐negative *C9orf72* test.

GRADE rating: A ‐ Strong.

Strength: This recommendation is supported by one study with Level 1 evidence.

**Recommendation 29a. Fragment size analysis of a PCR that spans the *C9orf72* repeat, either as a stand‐alone assay or as part of a dual‐mode PCR, is an acceptable method for sizing normal range alleles**.

*Key points*: Fragment size analysis is highly accurate at sizing normal and intermediate range *C9orf72* alleles, whereas standard Southern blot techniques are not. However, it should be noted that optimized Southern blot protocols, such as that described by Buchman et al. could enable accurate sizing of normal range *C9orf72* alleles by comparing cloned genomic fragments created by restriction enzyme digests at sites located within and close to the repeat expansion region.^89^

GRADE rating: B ‐ Moderate.

Strength: This recommendation is supported by one study with Level 2 evidence.

**Recommendation 29b. Expansion Hunter analysis of PCR‐free whole‐genome sequencing data alone is an acceptable method for sizing non‐expanded *C9orf72* alleles.**

*Key points*: The Expansion Hunter Software Tool developed and validated by Dolzhenko et al. shows high accuracy for normal *C9orf72* alleles when used on PCR‐free WGS short‐read data.^93^

GRADE rating: B ‐ Moderate.

Strength: This recommendation is supported by one study with Level 2 evidence.

**Recommendation 30. Testing reports for *C9orf72* should specify the sizes of non‐expanded alleles**.

*Key points*: There is currently no validated cutoff that differentiates between pathogenic and nonpathogenic alleles. The size of non‐expanded alleles should be documented on test reports in the event that alleles of a particular size are later determined to be unstable or confer increased disease risk.^10^, ^94^

GRADE rating: A ‐ Strong.

Strength: This recommendation is supported by at least 1 study with Level 1 evidence.

**Recommendation 31. Labs that classify *C9orf72* alleles as “intermediate” or “uncertain” should include a statement outlining up‐to‐date data regarding uncertainty of pathogenicity of these allele sizes.**

*Key points*: Currently, allele sizes of 20–29 repeats have contradictory evidence of association with ALS; if they do confer disease risk this is likely lower than for longer expansions. Laboratories reporting alleles classified as intermediate or uncertain should summarize current evidence, or lack of evidence, for pathogenicity of such alleles.^62^, ^94^

GRADE rating: A ‐ Strong.

Strength: This recommendation is supported by at least 1 study with Level 1 evidence.

**Recommendation 32. Labs reporting *C9orf72* repeat expansions should include a statement clearly outlining the maximum number of repeats detectable by the assay employed (e.g., >55 repeats; >145 repeats; 1500–2500 depending on the method).**

*Key points*: At this time, all commonly employed methods for detecting the presence of the *C9orf72* repeat expansion are unable to accurately determine the number of repeats. The upper boundary of the expansion size should be documented on test reports, given the possibility that expansions of a particular size could be shown in the future to confer specific disease risks or other clinical significance. For example, if we learn that pathogenicity begins at 90 repeats, a report designating a boundary of >55 repeats will allow clinicians to determine that retesting should be performed to determine if the patient has >90 repeats.

GRADE rating: D ‐ Expert.

Strength: There are no studies addressing this recommendation.

**Recommendation 33. The interrogation of non‐*C9orf72* ALS genes should utilize simultaneous sequencing methods (e.g., panel, exome, genome) rather than sequential gene sequencing.**

*Key points*: Simultaneous sequencing approaches, including multigene panel, whole‐exome or whole‐genome, reduce the cost and time to genetic diagnosis compared to sequential testing, and also enable identification of cases harboring pathogenic variants in more than one gene.^95^, ^96^, ^97^, ^98^ This recommendation may not apply in special situations (e.g., a familial variant is already known and can be assessed with Sanger sequencing of a single exon or where a patient's clinical phenotype is highly suggestive of a single gene).

GRADE rating: B ‐ Moderate.

Strength: This recommendation is supported by at least one study with Level 2 evidence.

**Recommendation 34. Based on ClinGen classifications, ALS gene panel reports should clearly differentiate between genes that are causal for ALS and those genes where the evidence is sparse, conflicting or insufficient**.

*Key points*: The strength of genetic evidence supporting the ability of specific genes to cause ALS varies widely and changes as additional studies are conducted. This evidence often shifts faster than the disease‐specific panels offered at testing laboratories and results in variants being reported for genes that are no longer considered monogenic causes of ALS. Laboratories should consider dropping these genes from their panels or at least designate the gene‐disease validity of tested genes, as classified by the Amyotrophic Lateral Sclerosis Spectrum Disorders Gene Curation Expert Panel of ClinGen (https://clinicalgenome.org/affiliation/40096/). In particular, genes classified as “Limited” or “Refuted” or not curated should be clearly differentiated on the report.

GRADE rating: D ‐ Expert.

Strength: There are no studies supporting this recommendation.

**Recommendation 35. When targeted‐capture, whole‐exome, or whole‐genome methods are used, gene regions that were not adequately assessed should be interrogated further or highlighted in the report.**

*Key points*: Due to difficulties generating adequate sequencing coverage or challenges with mapping and alignment, most current sequencing technologies have reduced sensitivity for variants in some genomic regions (e.g., exons 1 and 2 of *CHCHD10*). It is recommended that laboratories use additional methods to fill these gaps whenever possible. In the event that this is not performed, laboratory reports should clearly identify regions of genes where inadequate sequencing coverage or known issues may have decreased sensitivity for specific types of mutations. These specifications should aim to be interpretable by clinicians without genomic training (e.g., which amino acid stretches were missed rather than genomic coordinates or a percentage of base pairs for the entire exon or gene). This will enable ordering clinicians can accurately assess the likelihood of missed finding in their patients.

GRADE rating: D ‐ Expert.

Strength: There are no studies supporting this recommendation.

## Discussion

These evidence‐based, consensus guidelines for ALS genetic testing, counseling and methodologies are meant to establish a standard of care in clinical practice for individuals with ALS. Currently, persons with ALS in the United States have variable access to tertiary, academic, multidisciplinary ALS clinics staffed by genetic counselors. And while there has been improvement in access to multidisciplinary care clinics worldwide, the majority of people with ALS do not have access to such clinics.^99^, ^100^ These practice guidelines can be adopted by neurologists in private practice, in academic settings, and by other providers such as nurse practitioners and general practitioners when neurologists are not available. These guidelines will also serve these providers when genetic counselors are not available. Separate recommendations have been published for presymptomatic genetic counseling and testing.^57^

Genetic counselors in the United States are board certified by the American Board of Genetic Counseling and offer unique value in the care of individuals with ALS. They provide a perspective and training that neurologists and other medical providers do not typically have, including the treatment of family as the unit of care. These guidelines will support neurologists and other clinicians in providing genetic counseling when a board‐certified genetic counselor is not available and are intended to provide uniformity to the genetic counseling and testing approach of a person with ALS, regardless of clinical setting. Access to board‐certified genetic counselors is scarce, and the ideal scenario of an integrated genetic counselor within the multidisciplinary ALS care team is currently scarcer still. Given the rapidly increasing importance and need for genetic counselors in ALS, and in neurogenetics in general, research into alternative service delivery models is needed. Possible models to expand access to genetic counseling and patient education include telemedicine modalities, patient webinars with follow‐up genetic counseling, and online decision tools.^101^, ^102^

Nonetheless, genetic counselors will become increasingly required as part of multidisciplinary teams in neurology as the technological advances in our understanding of genetic risk and association progresses. In particular, the analysis and complexity of genetic testing results will necessitate expert interpretation and the ability to communicate complexity and uncertainty to patients and clinicians alike. The need for these specialized clinical skills will become ever more acute as gene‐targeted therapies are approved and patient eligibility is determined.

Our review of the literature on laboratory practices in commercial ALS genetic testing revealed a lack of consistency in methodologies and clinical reporting of results. It also revealed a paucity of published evidence for the interpretation and reporting of results. Commercial genetic testing laboratories establish their own workflows; however, the guidelines presented here are meant to serve as standards to harmonize the methodologies and reporting for these stakeholders.

These guidelines reflect current genomic technology, which will evolve along with our scientific understanding of the genetics of ALS. It is expected that genetic associations with ALS and related disorders will continue expand, and with it, the complexity of results that must be communicated. In the future, associations with the nonprotein coding genome are expected to advance for example, which will require increased genetics sophistication of ALS providers. We view these guidelines as a first step toward a uniform and equitable approach to ALS genetic testing that will require revision periodically as new genetic discoveries and new genetic therapies, both experimental and FDA‐approved, move forward for people living with ALS.

## Author Contributions

JR conceived and designed the study, acquired and analyzed data, and drafted the manuscript and figures. BE acquired and analyzed data and drafted figures. JW acquired and analyzed data. MH acquired and analyzed data and revised the manuscript. SK designed the study, acquired and analyzed data, and revised the manuscript and figures. The members of the Expert Panel (see Acknowledgments) reviewed the evidence summary, voted on each recommendation statement, and participated in a video conference to refine recommendations.

## Conflict of Interest

JR has served as a consultant for Biogen, Ionis, and Uniqure. MH has served as a consultant for Biogen, Amylyx, Invitae, Guidepoint Global, and MDA. BE, JW, and SK have no conflicts to declare.

## Supporting information


Table S1
