# Supplementary material for: Evidence‐based consensus guidelines for ALS genetic testing and counseling
Source: Ann Clin Transl Neurol. 2023 Sep 10;10(11):2074–91. doi: 10.1002/acn3.51895 (PMC10646996; doi:10.1002/acn3.51895)
Supplement: Supplementary file 1 — Table S1 [file ACN3-10-2074-s001.docx]

Evidence Summary Document

Genetic Testing Guidelines

# Recommendation 1: All pALS should be offered genetic testing.

**GRADE rating:** A - Strong

**Strength**: This recommendation is supported by at least one study of level 1 evidence.

**Included Studies**:

| **Authors and Publication Year** | **Title** | **Level of Evidence** |
| --- | --- | --- |
| Chen, 2021 | FUS mutation is probably the most common pathogenic gene for JALS, especially sporadic JALS | 1 |
| Chiò et al., 2018 | The multistep hypothesis of ALS revisited: The role of genetic mutations. | 1 |
| Debrayet al., 2013 | Frequency of C9orf72 repeat expansions in amyotrophic lateral sclerosis: a Belgian cohort study. | 1 |
| Grassano, et al., 2021 | Mutational Analysis of Known ALS Genes in an Italian Population-Based Cohort. | 1 |
| Kaivola et al., 2020 | Carriership of two copies of C9orf72 hexanucleotide repeat intermediate-length alleles is a risk factor for ALS in the Finnish population | 1 |
| Wei et al., 2019 | Unique characteristics of the genetics epidemiology of amyotrophic lateral sclerosis in China. | 1 |
| Zou et al., 2017 | Genetic epidemiology of amyotrophic lateral sclerosis: a systematic review and meta-analysis. | 1 |
| Beck et al., 2013 | Large C9orf72 hexanucleotide repeat expansions are seen in multiple neurodegenerative syndromes and are more frequent than expected in the UK population | 2 |
| Borghero et al., 2014 | Genetic architecture of ALS in Sardinia. | 2 |
| Cady et al., 2015 | Amyotrophic lateral sclerosis onset is influenced by the burden of rare variants in known amyotrophic lateral sclerosis genes. | 2 |
| Chen et al., 2015 | Large C9orf72 repeat expansions are seen in Chinese patients with sporadic amyotrophic lateral sclerosis. | 2 |
| Chen et al., 2020 | Clinical and genetic features of patients with amyotrophic lateral sclerosis in southern China. | 2 |
| Chen et al., 2020 | Role of genetics in amyotrophic lateral sclerosis: a large cohort study in Chinese mainland population | 2 |
| Chen et al., 2021 | Novel TARDBP missense mutation caused familial amyotrophic lateral sclerosis with frontotemporal dementia and parkinsonism. | 2 |
| Corrado et al., 2009 | High frequency of TARDBP gene mutations in Italian patients with amyotrophic lateral sclerosis. | 2 |
| Corrado et al., 2010 | Mutations of FUS gene in sporadic amyotrophic lateral sclerosis | 2 |
| Couthouis et al., 2014 | Targeted exon capture and sequencing in sporadic amyotrophic lateral sclerosis. | 2 |
| DeJesus-Hernandez et al., 2010 | De novo truncating FUS gene mutation as a cause of sporadic amyotrophic lateral sclerosis. | 2 |
| Erazo et al., 2022 | Epidemiological and genetic features of amyotrophic lateral sclerosis in Latin America and the Caribbean: a systematic review | 2 |
| Feng et al., 2021 | Genetic and clinical features of Chinese sporadic amyotrophic lateral sclerosis patients with TARDBP mutations | 2 |
| Gijselinck et al., 2012 | A C9orf72 promoter repeat expansion in a Flanders-Belgian cohort with disorders of the frontotemporal lobar degeneration-amyotrophic lateral sclerosis spectrum: A gene identification study | 2 |
| Gijselinck et al., 2016 | The C9orf72 repeat size correlates with onset age of disease, DNA methylation and transcriptional downregulation of the promoter. | 2 |
| Goldstein et al., 2017 | High frequency of C9orf72 hexanucleotide repeat expansion in amyotrophic lateral sclerosis patients from two founder populations sharing the same risk haplotype | 2 |
| Gromicho et al., 2018 | Frequency of C9orf72 hexanucleotide repeat expansion and SOD1 mutations in Portuguese patients with amyotrophic lateral sclerosis | 2 |
| Harms et al., 2013 | Lack of C9ORF72 coding mutations supports a gain of function for repeat expansions in amyotrophic lateral sclerosis | 2 |
| He et al., 2015 | C9orf72 hexanucleotide repeat expansions in Chinese sporadic amyotrophic lateral sclerosis | 2 |
| Hou et al., 2016 | Screening of SOD1, FUS and TARDBP genes in patients with amyotrophic lateral sclerosis in central-southern China. | 2 |
| Ju et al., 2016 | Two distinct clinical features and cognitive impairment in amyotrophic lateral sclerosis patients with TARDBP gene mutations in the Chinese population. | 2 |
| Keogh et al., 2017 | Genetic compendium of 1511 human brains available through the UK Medical Research Council Brain Banks Network Resource. | 2 |
| Kim et al., 2016 | Identification of mutations in Korean patients with amyotrophic lateral sclerosis using multigene panel testing. | 2 |
| Kwiatkowski et al., 2009 | Mutations in the FUS/TLS gene on chromosome 16 cause familial amyotrophic lateral sclerosis. | 2 |
| Lai et al., 2011 | FUS mutations in sporadic amyotrophic lateral sclerosis | 2 |
| Liu et al., 2016 | Mutation spectrum of Chinese patients with familial and sporadic amyotrophic lateral sclerosis. | 2 |
| Liu et al., 2021 | Mutation spectrum of amyotrophic lateral sclerosis in Central South China. | 2 |
| Majounie et al., 2012 | Frequency of the C9orf72 hexanucleotide repeat expansion in patients with amyotrophic lateral sclerosis and frontotemporal dementia: A cross-sectional study | 2 |
| Millecamps et al., 2010 | SOD1, ANG, VAPB, TARDBP, and FUS mutations in familial amyotrophic lateral sclerosis: Genotype-phenotype correlations | 2 |
| Millecamps et al., 2012 | Phenotype difference between ALS patients with expanded repeats in C9ORF72 and patients with mutations in other ALS-related genes. | 2 |
| Morgan et al., 2017 | A comprehensive analysis of rare genetic variation in amyotrophic lateral sclerosis in the UK. | 2 |
| Nakamura et al., 2016 | Next-generation sequencing of 28 ALS-related genes in a Japanese ALS cohort. | 2 |
| Nishiyama et al., 2017 | Comprehensive targeted next-generation sequencing in Japanese familial amyotrophic lateral sclerosis | 2 |
| Nordin et al., 2017 | Sequence variations in C9orf72 downstream of the hexanucleotide repeat region and its effect on repeat-primed PCR interpretation: a large multinational screening study | 2 |
| Özoguz et al., 2015 | The distinct genetic pattern of ALS in Turkey and novel mutations | 2 |
| Pensato et al., 2020 | Sorting rare ALS genetic variants by targeted re-sequencing panel in italian patients: OPTN, VCP, and SQSTM1 variants account for 3% of rare genetic forms | 2 |
| Rademakers et al., 2010 | FUS gene mutations in familial and sporadic amyotrophic lateral sclerosis | 2 |
| Ratti et al., 2012 | C9ORF72 repeat expansion in a large Italian ALS cohort: evidence of a founder effect. | 2 |
| Renton et al., 2011 | A hexanucleotide repeat expansion in C9ORF72 is the cause of chromosome 9p21-linked ALS-FTD. | 2 |
| Roggenbuck et al., 2020 | Incidence of pathogenic, likely pathogenic, and uncertain ALS variants in a clinic cohort | 2 |
| Rutherford et al., 2008 | Novel mutations in TARDBP(TDP-43) in patients with familial amyotrophic lateral sclerosis | 2 |
| Rutherford et al., 2012 | Pathogenicity of exonic indels in fused in sarcoma in amyotrophic lateral sclerosis. | 2 |
| Ryan et al., 2019 | Comparison of the clinical and genetic features of amyotrophic lateral sclerosis across Cuban, Uruguayan and Irish clinic-based populations. | 2 |
| Sabatelli et al., 2012 | C9ORF72 hexanucleotide repeat expansions in the Italian sporadic ALS population. | 2 |
| Sabatelli et al., 2013 | Mutations in the 3' untranslated region of FUS causing FUS overexpression are associated with amyotrophic lateral sclerosis. | 2 |
| Smith et al., 2013 | The C9ORF72 expansion mutation is a common cause of ALS+/-FTD in Europe and has a single founder. | 2 |
| Ticozzi et al., 2011 | Mutational analysis of TARDBP in neurodegenerative diseases. | 2 |
| Trojsi et al., 2019 | Comparative analysis of C9Orf72 and sporadic disease in a large multicenter ALS population: The effect of Male sex on survival of C9Orf72 positive patients | 2 |
| Tunca et al., 2020 | Revisiting the complex architecture of ALS in Turkey: Expanding genotypes, shared phenotypes, molecular networks, and a public variant database | 2 |
| Ungaro et al., 2021 | Genetic investigation of amyotrophic lateral sclerosis patients in south Italy: a two-decade analysis | 2 |
| Van Deerlin et al., 2008 | TARDBP mutations in amyotrophic lateral sclerosis with TDP-43 neuropathology: a genetic and histopathological analysis | 2 |
| Van Der Zee et al., 2012 | A pan-european study of the C9orf72 expansion associated with FTLD and ALS | 2 |
| van Rheenen et al., 2012 | Hexanucleotide repeat expansions in C9ORF72 in the spectrum of motor neuron diseases. | 2 |
| Waibel et al., 2013 | Truncating mutations in FUS/TLS give rise to a more aggressive ALS-phenotype than missense mutations: A clinico-genetic study in Germany | 2 |
| Wang et al., 2020 | Identification of novel FUS and TARDBP gene mutations in Chinese amyotrophic lateral sclerosis patients with HRM analysis. | 2 |
| Xi et al., 2012 | Investigation of C9orf72 in 4 neurodegenerative disorders | 2 |
| Yan et al., 2010 | Frameshift and novel mutations in FUS in familial amyotrophic lateral sclerosis and ALS/dementia | 2 |
| Zhang et al., 2018 | Screening for possible oligogenic pathogenesis in Chinese sporadic ALS patients. | 2 |
| Zou et al., 2012 | Screening of the FUS gene in familial and sporadic amyotrophic lateral sclerosis patients of Chinese origin. | 2 |
| Zou et al., 2021 | Novel FUS mutation Y526F causing rapidly progressive familial amyotrophic lateral sclerosis. | 2 |
| Abramycheva et al., 2015 | C9ORF72 hexanucleotide repeat expansion in ALS patients from the Central European Russia population | 3 |
| Belzil et al., 2009 | Mutations in FUS cause FALS and SALS in French and French Canadian populations. | 3 |
| Belzil et al., 2011 | Identification of novel FUS mutations in sporadic cases of amyotrophic lateral sclerosis. | 3 |
| Bertolin et al., 2014 | Improving the knowledge of amyotrophic lateral sclerosis genetics: Novel SOD1 and FUS variants | 3 |
| Byrne et al., 2012 | Cognitive and clinical characteristics of patients with amyotrophic lateral sclerosis carrying a C9orf72 repeat expansion: a population-based cohort study. | 3 |
| Chiò et al., 2011 | Large proportion of amyotrophic lateral sclerosis cases in sardinia due to a single founder mutation of the TARDBP gene | 3 |
| Chiò et al., 2012 | Extensive genetics of ALS : A population-based study in Italy | 3 |
| Chiò et al., 2012 | Clinical characteristics of patients with familial amyotrophic lateral sclerosis carrying the pathogenic GGGGCC hexanucleotide repeat expansion of C9ORF72. | 3 |
| Conforti et al., 2011 | TARDBP gene mutations in south Italian patients with amyotrophic lateral sclerosis. | 3 |
| Cooper-Knock et al., 2012 | Clinico-pathological features in amyotrophic lateral sclerosis with expansions in C9ORF72. | 3 |
| Czell et al., 2013 | Phenotypes in Swiss patients with familial ALS carrying TARDBP mutations | 3 |
| Del Bo et al., 2009. | TARDBP (TDP-43) sequence analysis in patients with familial and sporadic ALS: identification of two novel mutations. | 3 |
| Drepper et al., 2011 | C-terminal FUS/TLS mutations in familial and sporadic ALS in Germany | 3 |
| García-Redondo et al., 2013 | Analysis of the C9orf72 gene in patients with amyotrophic lateral sclerosis in Spain and different populations worldwide. | 3 |
| Gijselinck et al., 2009 | Neuronal inclusion protein TDP-43 has no primary genetic role in FTD and ALS | 3 |
| Gratten et al., 2017 | Whole-exome sequencing in amyotrophic lateral sclerosis suggests NEK1 is a risk gene in Chinese. | 3 |
| Hewitt et al., 2010 | Novel FUS/TLS mutations and pathology in familial and sporadic amyotrophic lateral sclerosis. | 3 |
| Hirano et al., 2018 | Noncoding repeat expansions for ALS in Japan are associated with the ATXN8OS gene | 3 |
| Huang et al., 2012 | TARDBP gene mutations among Chinese patients with sporadic amyotrophic lateral sclerosis | 3 |
| Jiao et al., 2014 | Identification of C9orf72 repeat expansions in patients with amyotrophic lateral sclerosis and frontotemporal dementia in mainland China. | 3 |
| Kamada et al., 2009 | Screening for TARDBP mutations in Japanese familial amyotrophic lateral sclerosis. | 3 |
| Kenna et al., 2013 | Delineating the genetic heterogeneity of ALS using targeted high-throughput sequencing | 3 |
| Kirby et al., 2010 | Broad clinical phenotypes associated with TAR-DNA binding protein (TARDBP) mutations in amyotrophic lateral sclerosis. | 3 |
| Konno et al., 2012 | Japanese amyotrophic lateral sclerosis patients with GGGGCC hexanucleotide repeat expansion in C9ORF72 | 3 |
| Kwon et al., 2012 | Screening of the SOD1, FUS, TARDBP, ANG, and OPTN mutations in Korean patients with familial and sporadic ALS. | 3 |
| Lattante et al., 2012 | Contribution of major amyotrophic lateral sclerosis genes to the etiology of sporadic disease | 3 |
| Liu et al., 2016 | Identification of a novel loss-of-function C9orf72 splice site mutation in a patient with amyotrophic lateral sclerosis. | 3 |
| Lysogorskaia et al., 2015 | Genetic studies of Russian patients with amyotrophic lateral sclerosis | 3 |
| Mok et al., 2012 | High frequency of the expanded C9ORF72 hexanucleotide repeat in familial and sporadic Greek ALS patients. | 3 |
| Narain et al., 2017 | C9orf72 hexanucleotide repeat expansions and Ataxin 2 intermediate length repeat expansions in Indian patients with amyotrophic lateral sclerosis | 3 |
| Narain et al., 2018 | Targeted next-generation sequencing reveals novel and rare variants in Indian patients with amyotrophic lateral sclerosis. | 3 |
| Ogaki et al., 2012 | Analysis of C9orf72 repeat expansion in 563 Japanese patients with amyotrophic lateral sclerosis | 3 |
| Shamim et al., 2020 | C9orf72 hexanucleotide repeat expansion in Indian patients with ALS: a common founder and its geographical predilection. | 3 |
| Soong et al., 2014 | Extensive molecular genetic survey of Taiwanese patients with amyotrophic lateral sclerosis. | 3 |
| Sproviero et al., 2012 | FUS mutations in sporadic amyotrophic lateral sclerosis: Clinical and genetic analysis | 3 |
| Tarlarini et al., 2015 | Novel FUS mutations identified through molecular screening in a large cohort of familial and sporadic amyotrophic lateral sclerosis. | 3 |
| Ticozzi et al., 2009 | Analysis of FUS gene mutation in familial amyotrophic lateral sclerosis within an Italian cohort. | 3 |
| Tsai et al., 2012 | A hexanucleotide repeat expansion in C9ORF72 causes familial and sporadic ALS in Taiwan. | 3 |
| van Blitterswijk et al., 2012 | Genetic overlap between apparently sporadic motor neuron diseases. | 3 |
| Vance et al., 2009 | Mutations in FUS, an RNA processing protein, cause familial amyotrophic lateral sclerosis type 6. | 3 |
| Williams et al., 2009 | A novel TARDBP mutation in an Australian amyotrophic lateral sclerosis kindred. | 3 |
| Ye et al., 2013 | Absence of Mutations in Exon 6 of the TARDBP Gene in 207 Chinese Patients with Sporadic Amyotrophic Lateral Sclerosis | 3 |
| Zou et al., 2013 | Screening for C9orf72 repeat expansions in Chinese amyotrophic lateral sclerosis patients | 3 |
| Zou et al., 2013 | De novo FUS gene mutations are associated with juvenile-onset sporadic amyotrophic lateral sclerosis in China | 3 |
| Zou et al., 2016 | The distinctive genetic architecture of ALS in mainland China | 3 |
| Akiyama et al., 2016 | Genotype-phenotype relationships in familial amyotrophic lateral sclerosis with FUS/TLS mutations in Japan | 4 |
| Alavi et al., 2014 | Repeat expansion in C9ORF72 is not a major cause of amyotrophic lateral sclerosis among iranian patients | 4 |
| Amador et al., 2021 | New advances in Amyotrophic Lateral Sclerosis genetics: Towards gene therapy opportunities for familial and young cases. | 4 |
| Arthur et al., 2017 | The Use of Genetic Testing in Amyotrophic Lateral Sclerosis by Neurologists | 4 |
| Blair et al., 2010 | FUS mutations in amyotrophic lateral sclerosis: clinical, pathological, neurophysiological and genetic analysis. | 4 |
| Boeve et al., 2012 | Characterization of frontotemporal dementia and/or amyotrophic lateral sclerosis associated with the GGGGCC repeat expansion in C9ORF72 | 4 |
| Borg et al., 2021 | Genetic analysis of ALS cases in the isolated island population of Malta | 4 |
| Brown et al., 2012 | SOD1, ANG, TARDBP and FUS mutations in amyotrophic lateral sclerosis: a United States clinical testing lab experience. | 4 |
| Chadi et al., 2017 | Genetic analyses of patients with familial and sporadic amyotrophic lateral sclerosis in a Brazilian research center | 4 |
| Chester et al., 2013 | Rapidly progressive frontotemporal dementia and bulbar amyotrophic lateral sclerosis in Portuguese patients with C9orf72 mutation. | 4 |
| Cintra et al., 2018 | The frequency of the C9orf72 expansion in a Brazilian population. | 4 |
| Conte et al., 2012 | Classification of familial amyotrophic lateral sclerosis by family history: Effects on frequency of genes mutation | 4 |
| Cooper-Knock et al., 2017 | Targeted Genetic Screen in Amyotrophic Lateral Sclerosis Reveals Novel Genetic Variants with Synergistic Effect on Clinical Phenotype. | 4 |
| Corcia et al., 2021 | Effect of familial clustering in the genetic screening of 235 French ALS families | 4 |
| Damme et al., 2010 | The occurrence of mutations in FUS in a Belgian cohort of patients with familial ALS. | 4 |
| Daria et al., 2019 | Genotypes of amyotrophic lateral sclerosis in Mongolia. | 4 |
| DeJesus-Hernandez et al., 2011 | Expanded GGGGCC Hexanucleotide Repeat in Noncoding Region of C9ORF72 Causes Chromosome 9p-Linked FTD and ALS | 4 |
| Dekker et al., 2016 | Large-scale screening in sporadic amyotrophic lateral sclerosis identifies genetic modifiers in C9orf72 repeat carriers. | 4 |
| Dobson-Stone et al., 2012 | C9ORF72 repeat expansion in clinical and neuropathologic frontotemporal dementia cohorts. | 4 |
| Dols-Icardo et al., 2018 | Analysis of known amyotrophic lateral sclerosis and frontotemporal dementia genes reveals a substantial genetic burden in patients manifesting both diseases not carrying the C9orf72 expansion mutation | 4 |
| Dombroski et al., 2013 | C9orf72 hexanucleotide repeat expansion and Guam amyotrophic lateral sclerosis-Parkinsonism-dementia complex | 4 |
| Edgar et al., 2021 | Mutation analysis of SOD1, C9orf72, TARDBP and FUS genes in ethnically-diverse Malaysian patients with amyotrophic lateral sclerosis (ALS). | 4 |
| Fratta et al., 2015 | Screening a UK amyotrophic lateral sclerosis cohort provides evidence of multiple origins of the C9orf72 expansion. | 4 |
| Gibson et al., 2017 | The evolving genetic risk for sporadic ALS | 4 |
| Gonçalves et al., 2021 | Genetic epidemiology of familial ALS in Brazil | 4 |
| Groen et al., 2010 | FUS mutations in familial amyotrophic lateral sclerosis in the Netherlands | 4 |
| Gromicho et al., 2020 | Targeted next-generation sequencing study in familial ALS-FTD Portuguese patients negative for C9orf72 HRE | 4 |
| Hübers et al., 2014 | Polymerase chain reaction and Southern blot-based analysis of the C9orf72 hexanucleotide repeat in different motor neuron diseases. | 4 |
| Hübers et al., 2015 | De novo FUS mutations are the most frequent genetic cause in early-onset German ALS patients | 4 |
| Ishiura et al., 2012 | C9ORF72 repeat expansion in amyotrophic lateral sclerosis in the Kii peninsula of Japan | 4 |
| Itzcovich et al., 2016 | Analysis of C9orf72 in patients with frontotemporal dementia and amyotrophic lateral sclerosis from Argentina. | 4 |
| Jang et al., 2013 | Analysis of the C9orf72 hexanucleotide repeat expansion in Korean patients with familial and sporadic amyotrophic lateral sclerosis | 4 |
| Krüger et al., 2016 | Rare Variants in Neurodegeneration Associated Genes Revealed by Targeted Panel Sequencing in a German ALS Cohort. | 4 |
| Lamp et al., 2018 | Twenty years of molecular analyses in amyotrophic lateral sclerosis: genetic landscape of Italian patients | 4 |
| Liu et al., 2013 | C9orf72 repeat expansions are not detected in Chinese patients with familial ALS. | 4 |
| Liu et al., 2014 | Identify mutation in amyotrophic lateral sclerosis cases using HaloPlex target enrichment system. | 4 |
| Liu et al., 2017 | The investigation of genetic and clinical features in Chinese patients with juvenile amyotrophic lateral sclerosis | 4 |
| Liu et al., 2019 | Genetic spectrum and variability in Chinese patients with amyotrophic lateral sclerosis | 4 |
| Luquin et al., 2009 | Genetic variants in the promoter of TARDBP in sporadic amyotrophic lateral sclerosis. | 4 |
| Ma et al., 2022 | Genetic analysis in Chinese patients with familial or young-onset amyotrophic lateral sclerosis | 4 |
| Marjanović et al., 2017 | Comparison of the clinical and cognitive features of genetically positive ALS patients from the largest tertiary center in Serbia. | 4 |
| McCann et al., 2017 | The genotype-phenotype landscape of familial amyotrophic lateral sclerosis in Australia | 4 |
| McCluskey et al., 2014 | ALS-Plus syndrome: non-pyramidal features in a large ALS cohort. | 4 |
| Mentula et al., 2012 | TARDBP mutations are not a frequent cause of ALS in Finnish patients | 4 |
| Mesaros et al., 2021 | Investigating the Genetic Profile of the Amyotrophic Lateral Sclerosis/Frontotemporal Dementia (ALS-FTD) Continuum in Patients of Diverse Race, Ethnicity and Ancestry | 4 |
| Morgan et al., 2015 | Investigation of next-generation sequencing technologies as a diagnostic tool for amyotrophic lateral sclerosis | 4 |
| Müller et al., 2018 | Comprehensive analysis of the mutation spectrum in 301 German ALS families. | 4 |
| Naruse et al., 2018 | Molecular epidemiological study of familial amyotrophic lateral sclerosis in Japanese population by whole-exome sequencing and identification of novel HNRNPA1 mutation. | 4 |
| Naruse et al., 2019 | Burden of rare variants in causative genes for amyotrophic lateral sclerosis (ALS) accelerates age at onset of ALS | 4 |
| Nel et al., 2019 | C9orf72 repeat expansions in South Africans with amyotrophic lateral sclerosis. | 4 |
| Origone et al., 2010 | Enlarging clinical spectrum of FALS with TARDBP gene mutations: S393L variant in an Italian family showing phenotypic variability and relevance for genetic counseling. | 4 |
| Orrù et al., 2012 | High frequency of the TARDBP p.Ala382Thr mutation in Sardinian patients with amyotrophic lateral sclerosis. | 4 |
| Pamphlett et al., 2012 | Transmission of C9orf72 hexanucleotide repeat expansions in sporadic amyotrophic lateral sclerosis: An Australian trio study | 4 |
| Pang et al., 2017 | Burden of rare variants in ALS genes influences survival in familial and sporadic ALS. | 4 |
| Piaceri et al., 2012 | Clinical heterogeneity in Italian patients with amyotrophic lateral sclerosis. | 4 |
| Roggenbuck et al., 2021 | Amyotrophic Lateral Sclerosis Genetic Access Program: Paving the Way for Genetic Characterization of ALS in the Clinic. | 4 |
| Scarlino et al., 2020 | Burden of rare variants in ALS and axonal hereditary neuropathy genes influence survival in ALS: Insights from a next generation sequencing study of an Italian ALS cohort | 4 |
| Scotter et al., 2017 | C9ORF72 and UBQLN2 mutations are causes of amyotrophic lateral sclerosis in New Zealand: a genetic and pathologic study using banked human brain tissue. | 4 |
| Simón-Sánchez et al., 2012 | The clinical and pathological phenotype of C9ORF72 hexanucleotide repeat expansions. | 4 |
| Sokratous et al., 2020 | Prevalence of C9orf72 hexanucleotide repeat expansion in Greek patients with sporadic ALS | 4 |
| Solski et al., 2012 | A novel TARDBP insertion/deletion mutation in the flail arm variant of amyotrophic lateral sclerosis | 4 |
| Stewart et al., 2012 | Clinical and pathological features of amyotrophic lateral sclerosis caused by mutation in the C9ORF72 gene on chromosome 9p | 4 |
| Sun et al., 2020 | Study on sleep-wake disorders in patients with genetic and non-genetic amyotrophic lateral sclerosis. | 4 |
| Syriani et al., 2011 | FUS/TLS gene mutations are the second most frequent cause of familial ALS in the Spanish population | 4 |
| Tripolszki et al., 2017 | Genetic analysis of the SOD1 and C9ORF72 genes in Hungarian patients with amyotrophic lateral sclerosis. | 4 |
| Tripolszki et al., 2019 | Comprehensive Genetic Analysis of a Hungarian Amyotrophic Lateral Sclerosis Cohort. | 4 |
| Tsai et al., 2013 | FUS, TARDBP, and SOD1 mutations in a Taiwanese cohort with familial ALS | 4 |
| Umoh et al., 2016 | Comparative analysis of C9orf72 and sporadic disease in an ALS clinic population | 4 |
| van Blitterswijk et al., 2012 | Evidence for an oligogenic basis of amyotrophic lateral sclerosis. | 4 |
| Van Langenhove et al., 2010 | Genetic contribution of FUS to frontotemporal lobar degeneration. | 4 |
| Vats et al., 2017 | Analysis of C9orf72 repeat expansion in amyotrophic lateral sclerosis patients from North India. | 4 |
| Vrabec et al., 2015 | Genetic analysis of amyotrophic lateral sclerosis in the Slovenian population. | 4 |
| Waibel et al., 2010 | Novel missense and truncating mutations in FUS/TLS in familial ALS. | 4 |
| Williams et al., 2013 | Pathophysiological insights into ALS with C9ORF72 expansions. | 4 |
| Xiong et al., 2010 | Association between novel TARDBP mutations and Chinese patients with amyotrophic lateral sclerosis. | 4 |
| Xu et al., 2018 | High frequency of the TARDBP p.M337Â V mutation among south-eastern Chinese patients with familial amyotrophic lateral sclerosis. | 4 |
| Zou et al., 2012 | Screening of the TARDBP gene in familial and sporadic amyotrophic lateral sclerosis patients of Chinese origin. | 4 |
| Zou et al., 2016 | Mutations in FUS are the most frequent genetic cause in juvenile sporadic ALS patients of Chinese origin | 4 |
| Salmon et al., 2022 | The importance of offering early genetic testing in everyone with amyotrophic lateral sclerosis | 5 |

**Recommendation 2: All pALS should be offered testing with an ALS gene panel that includes C9.**

**GRADE rating:** A - Strong

**Strength**: This recommendation is supported by at least one study of level 1 evidence.

**Included studies**:

| **Authors and Publication Year** | **Title** | **Level of Evidence** |
| --- | --- | --- |
| Chen, 2021 | FUS mutation is probably the most common pathogenic gene for JALS, especially sporadic JALS | 1 |
| Chiò et al., 2018 | The multistep hypothesis of ALS revisited: The role of genetic mutations. | 1 |
| Debrayet al., 2013 | Frequency of C9orf72 repeat expansions in amyotrophic lateral sclerosis: a Belgian cohort study. | 1 |
| Grassano, et al., 2021 | Mutational Analysis of Known ALS Genes in an Italian Population-Based Cohort. | 1 |
| Kaivola et al., 2020 | Carriership of two copies of C9orf72 hexanucleotide repeat intermediate-length alleles is a risk factor for ALS in the Finnish population | 1 |
| Wei et al., 2019 | Unique characteristics of the genetics epidemiology of amyotrophic lateral sclerosis in China. | 1 |
| Zou et al., 2017 | Genetic epidemiology of amyotrophic lateral sclerosis: a systematic review and meta-analysis. | 1 |
| Beck et al., 2013 | Large C9orf72 hexanucleotide repeat expansions are seen in multiple neurodegenerative syndromes and are more frequent than expected in the UK population | 2 |
| Borghero et al., 2014 | Genetic architecture of ALS in Sardinia. | 2 |
| Cady et al., 2015 | Amyotrophic lateral sclerosis onset is influenced by the burden of rare variants in known amyotrophic lateral sclerosis genes. | 2 |
| Chen et al., 2020 | Role of genetics in amyotrophic lateral sclerosis: a large cohort study in Chinese mainland population | 2 |
| Chen et al., 2015 | Large C9orf72 repeat expansions are seen in Chinese patients with sporadic amyotrophic lateral sclerosis. | 2 |
| Chen et al., 2020 | Clinical and genetic features of patients with amyotrophic lateral sclerosis in southern China. | 2 |
| Couthouis et al., 2014 | Targeted exon capture and sequencing in sporadic amyotrophic lateral sclerosis. | 2 |
| DeJesus-Hernandez et al., 2010 | De novo truncating FUS gene mutation as a cause of sporadic amyotrophic lateral sclerosis. | 2 |
| Erazo et al., 2022 | Epidemiological and genetic features of amyotrophic lateral sclerosis in Latin America and the Caribbean: a systematic review | 2 |
| Gijselinck et al., 2012 | A C9orf72 promoter repeat expansion in a Flanders-Belgian cohort with disorders of the frontotemporal lobar degeneration-amyotrophic lateral sclerosis spectrum: A gene identification study | 2 |
| Gijselinck et al., 2016 | The C9orf72 repeat size correlates with onset age of disease, DNA methylation and transcriptional downregulation of the promoter. | 2 |
| Goldstein et al., 2017 | High frequency of C9orf72 hexanucleotide repeat expansion in amyotrophic lateral sclerosis patients from two founder populations sharing the same risk haplotype | 2 |
| Gromicho et al., 2018 | Frequency of C9orf72 hexanucleotide repeat expansion and SOD1 mutations in Portuguese patients with amyotrophic lateral sclerosis | 2 |
| Harms et al., 2013 | Lack of C9ORF72 coding mutations supports a gain of function for repeat expansions in amyotrophic lateral sclerosis | 2 |
| He et al., 2015 | C9orf72 hexanucleotide repeat expansions in Chinese sporadic amyotrophic lateral sclerosis | 2 |
| Hou et al., 2016 | Screening of SOD1, FUS and TARDBP genes in patients with amyotrophic lateral sclerosis in central-southern China. | 2 |
| Keogh et al., 2017 | Genetic compendium of 1511 human brains available through the UK Medical Research Council Brain Banks Network Resource. | 2 |
| Kim et al., 2016 | Identification of mutations in Korean patients with amyotrophic lateral sclerosis using multigene panel testing. | 2 |
| Liu et al., 2016 | Mutation spectrum of Chinese patients with familial and sporadic amyotrophic lateral sclerosis. | 2 |
| Liu et al., 2021 | Mutation spectrum of amyotrophic lateral sclerosis in Central South China. | 2 |
| Majounie et al., 2012 | Frequency of the C9orf72 hexanucleotide repeat expansion in patients with amyotrophic lateral sclerosis and frontotemporal dementia: A cross-sectional study | 2 |
| Millecamps et al., 2010 | SOD1, ANG, VAPB, TARDBP, and FUS mutations in familial amyotrophic lateral sclerosis: Genotype-phenotype correlations | 2 |
| Millecamps et al., 2012 | Phenotype difference between ALS patients with expanded repeats in C9ORF72 and patients with mutations in other ALS-related genes. | 2 |
| Morgan et al., 2017 | A comprehensive analysis of rare genetic variation in amyotrophic lateral sclerosis in the UK. | 2 |
| Nakamura et al., 2016 | Next-generation sequencing of 28 ALS-related genes in a Japanese ALS cohort. | 2 |
| Nishiyama et al., 2017 | Comprehensive targeted next-generation sequencing in Japanese familial amyotrophic lateral sclerosis | 2 |
| Nordin et al., 2017 | Sequence variations in C9orf72 downstream of the hexanucleotide repeat region and its effect on repeat-primed PCR interpretation: a large multinational screening study | 2 |
| Özoguz et al., 2015 | The distinct genetic pattern of ALS in Turkey and novel mutations | 2 |
| Pensato et al., 2020 | Sorting rare ALS genetic variants by targeted re-sequencing panel in italian patients: OPTN, VCP, and SQSTM1 variants account for 3% of rare genetic forms | 2 |
| Ratti et al., 2012 | C9ORF72 repeat expansion in a large Italian ALS cohort: evidence of a founder effect. | 2 |
| Renton et al., 2011 | A hexanucleotide repeat expansion in C9ORF72 is the cause of chromosome 9p21-linked ALS-FTD. | 2 |
| Roggenbuck et al., 2020 | Incidence of pathogenic, likely pathogenic, and uncertain ALS variants in a clinic cohort | 2 |
| Ryan et al., 2019 | Comparison of the clinical and genetic features of amyotrophic lateral sclerosis across Cuban, Uruguayan and Irish clinic-based populations. | 2 |
| Sabatelli et al., 2012 | C9ORF72 hexanucleotide repeat expansions in the Italian sporadic ALS population. | 2 |
| Smith et al., 2013 | The C9ORF72 expansion mutation is a common cause of ALS+/-FTD in Europe and has a single founder. | 2 |
| Trojsi et al., 2019 | Comparative analysis of C9Orf72 and sporadic disease in a large multicenter ALS population: The effect of Male sex on survival of C9Orf72 positive patients | 2 |
| Tunca et al., 2020 | Revisiting the complex architecture of ALS in Turkey: Expanding genotypes, shared phenotypes, molecular networks, and a public variant database | 2 |
| Ungaro et al., 2021 | Genetic investigation of amyotrophic lateral sclerosis patients in south Italy: a two-decade analysis | 2 |
| Van Der Zee et al., 2012 | A pan-european study of the C9orf72 expansion associated with FTLD and ALS | 2 |
| van Rheenen et al., 2012 | Hexanucleotide repeat expansions in C9ORF72 in the spectrum of motor neuron diseases. | 2 |
| Xi et al., 2012 | Investigation of C9orf72 in 4 neurodegenerative disorders | 2 |
| Zhang et al., 2018 | Screening for possible oligogenic pathogenesis in Chinese sporadic ALS patients. | 2 |
| Abramycheva et al., 2015 | C9ORF72 hexanucleotide repeat expansion in ALS patients from the Central European Russia population | 3 |
| Bertolin et al., 2014 | Improving the knowledge of amyotrophic lateral sclerosis genetics: Novel SOD1 and FUS variants | 3 |
| Byrne et al., 2012 | Cognitive and clinical characteristics of patients with amyotrophic lateral sclerosis carrying a C9orf72 repeat expansion: a population-based cohort study. | 3 |
| Chiò et al., 2012 | Extensive genetics of ALS : A population-based study in Italy | 3 |
| Chiò et al., 2012 | Clinical characteristics of patients with familial amyotrophic lateral sclerosis carrying the pathogenic GGGGCC hexanucleotide repeat expansion of C9ORF72. | 3 |
| Cooper-Knock et al., 2012 | Clinico-pathological features in amyotrophic lateral sclerosis with expansions in C9ORF72. | 3 |
| García-Redondo et al., 2013 | Analysis of the C9orf72 gene in patients with amyotrophic lateral sclerosis in Spain and different populations worldwide. | 3 |
| Gratten et al., 2017 | Whole-exome sequencing in amyotrophic lateral sclerosis suggests NEK1 is a risk gene in Chinese. | 3 |
| Hirano et al., 2018 | Noncoding repeat expansions for ALS in Japan are associated with the ATXN8OS gene | 3 |
| Jiao et al., 2014 | Identification of C9orf72 repeat expansions in patients with amyotrophic lateral sclerosis and frontotemporal dementia in mainland China. | 3 |
| Kenna et al., 2013 | Delineating the genetic heterogeneity of ALS using targeted high-throughput sequencing | 3 |
| Konno et al., 2012 | Japanese amyotrophic lateral sclerosis patients with GGGGCC hexanucleotide repeat expansion in C9ORF72 | 3 |
| Kwon et al., 2012 | Screening of the SOD1, FUS, TARDBP, ANG, and OPTN mutations in Korean patients with familial and sporadic ALS. | 3 |
| Lattante et al., 2012 | Contribution of major amyotrophic lateral sclerosis genes to the etiology of sporadic disease | 3 |
| Lysogorskaia et al., 2015 | Genetic studies of Russian patients with amyotrophic lateral sclerosis | 3 |
| Mok et al., 2012 | High frequency of the expanded C9ORF72 hexanucleotide repeat in familial and sporadic Greek ALS patients. | 3 |
| Narain et al., 2017 | C9orf72 hexanucleotide repeat expansions and Ataxin 2 intermediate length repeat expansions in Indian patients with amyotrophic lateral sclerosis | 3 |
| Narain et al., 2018 | Targeted next-generation sequencing reveals novel and rare variants in Indian patients with amyotrophic lateral sclerosis. | 3 |
| Ogaki et al., 2012 | Analysis of C9orf72 repeat expansion in 563 Japanese patients with amyotrophic lateral sclerosis | 3 |
| Shamim et al., 2020 | C9orf72 hexanucleotide repeat expansion in Indian patients with ALS: a common founder and its geographical predilection. | 3 |
| Soong et al., 2014 | Extensive molecular genetic survey of Taiwanese patients with amyotrophic lateral sclerosis. | 3 |
| Tsai et al., 2012 | A hexanucleotide repeat expansion in C9ORF72 causes familial and sporadic ALS in Taiwan. | 3 |
| van Blitterswijk et al., 2012 | Genetic overlap between apparently sporadic motor neuron diseases. | 3 |
| Zou et al., 2013 | Screening for C9orf72 repeat expansions in Chinese amyotrophic lateral sclerosis patients | 3 |
| Zou et al., 2016 | The distinctive genetic architecture of ALS in mainland China | 3 |
| Alavi et al., 2014 | Repeat expansion in C9ORF72 is not a major cause of amyotrophic lateral sclerosis among iranian patients | 4 |
| Amador et al., 2021 | New advances in Amyotrophic Lateral Sclerosis genetics: Towards gene therapy opportunities for familial and young cases. | 4 |
| Arthur et al., 2017 | The Use of Genetic Testing in Amyotrophic Lateral Sclerosis by Neurologists | 4 |
| Boeve et al., 2012 | Characterization of frontotemporal dementia and/or amyotrophic lateral sclerosis associated with the GGGGCC repeat expansion in C9ORF72 | 4 |
| Borg et al., 2021 | Genetic analysis of ALS cases in the isolated island population of Malta | 4 |
| Brown et al., 2012 | SOD1, ANG, TARDBP and FUS mutations in amyotrophic lateral sclerosis: a United States clinical testing lab experience. | 4 |
| Chadi et al., 2017 | Genetic analyses of patients with familial and sporadic amyotrophic lateral sclerosis in a Brazilian research center | 4 |
| Chester et al., 2013 | Rapidly progressive frontotemporal dementia and bulbar amyotrophic lateral sclerosis in Portuguese patients with C9orf72 mutation. | 4 |
| Cintra et al., 2018 | The frequency of the C9orf72 expansion in a Brazilian population. | 4 |
| Conte et al., 2012 | Classification of familial amyotrophic lateral sclerosis by family history: Effects on frequency of genes mutation | 4 |
| Cooper-Knock et al., 2017 | Targeted Genetic Screen in Amyotrophic Lateral Sclerosis Reveals Novel Genetic Variants with Synergistic Effect on Clinical Phenotype. | 4 |
| Corcia et al., 2021 | Effect of familial clustering in the genetic screening of 235 French ALS families | 4 |
| Daria et al., 2019 | Genotypes of amyotrophic lateral sclerosis in Mongolia. | 4 |
| DeJesus-Hernandez et al., 2011 | Expanded GGGGCC Hexanucleotide Repeat in Noncoding Region of C9ORF72 Causes Chromosome 9p-Linked FTD and ALS | 4 |
| Dekker et al., 2016 | Large-scale screening in sporadic amyotrophic lateral sclerosis identifies genetic modifiers in C9orf72 repeat carriers. | 4 |
| Dobson-Stone et al., 2012 | C9ORF72 repeat expansion in clinical and neuropathologic frontotemporal dementia cohorts. | 4 |
| Dols-Icardo et al., 2018 | Analysis of known amyotrophic lateral sclerosis and frontotemporal dementia genes reveals a substantial genetic burden in patients manifesting both diseases not carrying the C9orf72 expansion mutation | 4 |
| Dombroski et al., 2013 | C9orf72 hexanucleotide repeat expansion and Guam amyotrophic lateral sclerosis-Parkinsonism-dementia complex | 4 |
| Edgar et al., 2021 | Mutation analysis of SOD1, C9orf72, TARDBP and FUS genes in ethnically-diverse Malaysian patients with amyotrophic lateral sclerosis (ALS). | 4 |
| Fratta et al., 2015 | Screening a UK amyotrophic lateral sclerosis cohort provides evidence of multiple origins of the C9orf72 expansion. | 4 |
| Gibson et al., 2017 | The evolving genetic risk for sporadic ALS | 4 |
| Gonçalves et al., 2021 | Genetic epidemiology of familial ALS in Brazil | 4 |
| Gromicho et al., 2020 | Targeted next-generation sequencing study in familial ALS-FTD Portuguese patients negative for C9orf72 HRE | 4 |
| Hübers et al., 2014 | Polymerase chain reaction and Southern blot-based analysis of the C9orf72 hexanucleotide repeat in different motor neuron diseases. | 4 |
| Ishiura et al., 2012 | C9ORF72 repeat expansion in amyotrophic lateral sclerosis in the Kii peninsula of Japan | 4 |
| Itzcovich et al., 2016 | Analysis of C9orf72 in patients with frontotemporal dementia and amyotrophic lateral sclerosis from Argentina. | 4 |
| Jang et al., 2013 | Analysis of the C9orf72 hexanucleotide repeat expansion in Korean patients with familial and sporadic amyotrophic lateral sclerosis | 4 |
| Krüger et al., 2016 | Rare Variants in Neurodegeneration Associated Genes Revealed by Targeted Panel Sequencing in a German ALS Cohort. | 4 |
| Lamp et al., 2018 | Twenty years of molecular analyses in amyotrophic lateral sclerosis: genetic landscape of Italian patients | 4 |
| Liu et al., 2013 | C9orf72 repeat expansions are not detected in Chinese patients with familial ALS. | 4 |
| Liu et al., 2014 | Identify mutation in amyotrophic lateral sclerosis cases using HaloPlex target enrichment system. | 4 |
| Liu et al., 2017 | The investigation of genetic and clinical features in Chinese patients with juvenile amyotrophic lateral sclerosis | 4 |
| Liu et al., 2019 | Genetic spectrum and variability in Chinese patients with amyotrophic lateral sclerosis | 4 |
| Ma et al., 2022 | Genetic analysis in Chinese patients with familial or young-onset amyotrophic lateral sclerosis | 4 |
| Marjanović et al., 2017 | Comparison of the clinical and cognitive features of genetically positive ALS patients from the largest tertiary center in Serbia. | 4 |
| McCann et al., 2017 | The genotype-phenotype landscape of familial amyotrophic lateral sclerosis in Australia | 4 |
| McCluskey et al., 2014 | ALS-Plus syndrome: non-pyramidal features in a large ALS cohort. | 4 |
| Mesaros et al., 2021 | Investigating the Genetic Profile of the Amyotrophic Lateral Sclerosis/Frontotemporal Dementia (ALS-FTD) Continuum in Patients of Diverse Race, Ethnicity and Ancestry | 4 |
| Morgan et al., 2015 | Investigation of next-generation sequencing technologies as a diagnostic tool for amyotrophic lateral sclerosis | 4 |
| Müller et al., 2018 | Comprehensive analysis of the mutation spectrum in 301 German ALS families. | 4 |
| Naruse et al., 2018 | Molecular epidemiological study of familial amyotrophic lateral sclerosis in Japanese population by whole-exome sequencing and identification of novel HNRNPA1 mutation. | 4 |
| Naruse et al., 2019 | Burden of rare variants in causative genes for amyotrophic lateral sclerosis (ALS) accelerates age at onset of ALS | 4 |
| Pamphlett et al., 2012 | Transmission of C9orf72 hexanucleotide repeat expansions in sporadic amyotrophic lateral sclerosis: An Australian trio study | 4 |
| Pang et al., 2017 | Burden of rare variants in ALS genes influences survival in familial and sporadic ALS. | 4 |
| Piaceri et al., 2012 | Clinical heterogeneity in Italian patients with amyotrophic lateral sclerosis. | 4 |
| Roggenbuck et al., 2021 | Amyotrophic Lateral Sclerosis Genetic Access Program: Paving the Way for Genetic Characterization of ALS in the Clinic. | 4 |
| Scarlino et al., 2020 | Burden of rare variants in ALS and axonal hereditary neuropathy genes influence survival in ALS: Insights from a next generation sequencing study of an Italian ALS cohort | 4 |
| Scotter et al., 2017 | C9ORF72 and UBQLN2 mutations are causes of amyotrophic lateral sclerosis in New Zealand: a genetic and pathologic study using banked human brain tissue. | 4 |
| Simón-Sánchez et al., 2012 | The clinical and pathological phenotype of C9ORF72 hexanucleotide repeat expansions. | 4 |
| Sokratous et al., 2020 | Prevalence of C9orf72 hexanucleotide repeat expansion in Greek patients with sporadic ALS | 4 |
| Stewart et al., 2012 | Clinical and pathological features of amyotrophic lateral sclerosis caused by mutation in the C9ORF72 gene on chromosome 9p | 4 |
| Sun et al., 2020 | Study on sleep-wake disorders in patients with genetic and non-genetic amyotrophic lateral sclerosis. | 4 |
| Tripolszki et al., 2017 | Genetic analysis of the SOD1 and C9ORF72 genes in Hungarian patients with amyotrophic lateral sclerosis. | 4 |
| Tripolszki et al., 2019 | Comprehensive Genetic Analysis of a Hungarian Amyotrophic Lateral Sclerosis Cohort. | 4 |
| Tsai et al., 2013 | FUS, TARDBP, and SOD1 mutations in a Taiwanese cohort with familial ALS | 4 |
| Umoh et al., 2016 | Comparative analysis of C9orf72 and sporadic disease in an ALS clinic population | 4 |
| van Blitterswijk et al., 2012 | Evidence for an oligogenic basis of amyotrophic lateral sclerosis. | 4 |
| Vats et al., 2017 | Analysis of C9orf72 repeat expansion in amyotrophic lateral sclerosis patients from North India. | 4 |
| Vrabec et al., 2015 | Genetic analysis of amyotrophic lateral sclerosis in the Slovenian population. | 4 |
| Williams et al., 2013 | Pathophysiological insights into ALS with C9ORF72 expansions. | 4 |
| Salmon et al., 2022 | The importance of offering early genetic testing in everyone with amyotrophic lateral sclerosis | 5 |

**Recommendation 3: All pALS should be offered testing with an ALS gene panel that includes *SOD1.***

**GRADE rating:** A - Strong

**Strength**: This recommendation is supported by at least one study of level 1 evidence.

**Included studies**:

| **Authors and Publication Year** | **Title** | **Level of Evidence** |
| --- | --- | --- |
| Chiò et al., 2018 | The multistep hypothesis of ALS revisited: The role of genetic mutations. | 1 |
| Grassano, et al., 2021 | Mutational Analysis of Known ALS Genes in an Italian Population-Based Cohort. | 1 |
| Wei et al., 2019 | Unique characteristics of the genetics epidemiology of amyotrophic lateral sclerosis in China. | 1 |
| Zou et al., 2017 | Genetic epidemiology of amyotrophic lateral sclerosis: a systematic review and meta-analysis. | 1 |
| Borghero et al., 2014 | Genetic architecture of ALS in Sardinia. | 2 |
| Cady et al., 2015 | Amyotrophic lateral sclerosis onset is influenced by the burden of rare variants in known amyotrophic lateral sclerosis genes. | 2 |
| Chen et al., 2020 | Role of genetics in amyotrophic lateral sclerosis: a large cohort study in Chinese mainland population | 2 |
| Chen et al., 2020 | Clinical and genetic features of patients with amyotrophic lateral sclerosis in southern China. | 2 |
| Couthouis et al., 2014 | Targeted exon capture and sequencing in sporadic amyotrophic lateral sclerosis. | 2 |
| DeJesus-Hernandez et al., 2010 | De novo truncating FUS gene mutation as a cause of sporadic amyotrophic lateral sclerosis. | 2 |
| Erazo et al., 2022 | Epidemiological and genetic features of amyotrophic lateral sclerosis in Latin America and the Caribbean: a systematic review | 2 |
| Gromicho et al., 2018 | Frequency of C9orf72 hexanucleotide repeat expansion and SOD1 mutations in Portuguese patients with amyotrophic lateral sclerosis | 2 |
| Hou et al., 2016 | Screening of SOD1, FUS and TARDBP genes in patients with amyotrophic lateral sclerosis in central-southern China. | 2 |
| Keogh et al., 2017 | Genetic compendium of 1511 human brains available through the UK Medical Research Council Brain Banks Network Resource. | 2 |
| Kim et al., 2016 | Identification of mutations in Korean patients with amyotrophic lateral sclerosis using multigene panel testing. | 2 |
| Liu et al., 2016 | Mutation spectrum of Chinese patients with familial and sporadic amyotrophic lateral sclerosis. | 2 |
| Liu et al., 2021 | Mutation spectrum of amyotrophic lateral sclerosis in Central South China. | 2 |
| Millecamps et al., 2010 | SOD1, ANG, VAPB, TARDBP, and FUS mutations in familial amyotrophic lateral sclerosis: Genotype-phenotype correlations | 2 |
| Morgan et al., 2017 | A comprehensive analysis of rare genetic variation in amyotrophic lateral sclerosis in the UK. | 2 |
| Nakamura et al., 2016 | Next-generation sequencing of 28 ALS-related genes in a Japanese ALS cohort. | 2 |
| Nishiyama et al., 2017 | Comprehensive targeted next-generation sequencing in Japanese familial amyotrophic lateral sclerosis | 2 |
| Özoguz et al., 2015 | The distinct genetic pattern of ALS in Turkey and novel mutations | 2 |
| Pensato et al., 2020 | Sorting rare ALS genetic variants by targeted re-sequencing panel in italian patients: OPTN, VCP, and SQSTM1 variants account for 3% of rare genetic forms | 2 |
| Roggenbuck et al., 2020 | Incidence of pathogenic, likely pathogenic, and uncertain ALS variants in a clinic cohort | 2 |
| Ryan et al., 2019 | Comparison of the clinical and genetic features of amyotrophic lateral sclerosis across Cuban, Uruguayan and Irish clinic-based populations. | 2 |
| Tunca et al., 2020 | Revisiting the complex architecture of ALS in Turkey: Expanding genotypes, shared phenotypes, molecular networks, and a public variant database | 2 |
| Ungaro et al., 2021 | Genetic investigation of amyotrophic lateral sclerosis patients in south Italy: a two-decade analysis | 2 |
| Zhang et al., 2018 | Screening for possible oligogenic pathogenesis in Chinese sporadic ALS patients. | 2 |
| Bertolin et al., 2014 | Improving the knowledge of amyotrophic lateral sclerosis genetics: Novel SOD1 and FUS variants | 3 |
| Byrne et al., 2012 | Cognitive and clinical characteristics of patients with amyotrophic lateral sclerosis carrying a C9orf72 repeat expansion: a population-based cohort study. | 3 |
| Chiò et al., 2012 | Extensive genetics of ALS : A population-based study in Italy | 3 |
| Gratten et al., 2017 | Whole-exome sequencing in amyotrophic lateral sclerosis suggests NEK1 is a risk gene in Chinese. | 3 |
| Kenna et al., 2013 | Delineating the genetic heterogeneity of ALS using targeted high-throughput sequencing | 3 |
| Kwon et al., 2012 | Screening of the SOD1, FUS, TARDBP, ANG, and OPTN mutations in Korean patients with familial and sporadic ALS. | 3 |
| Lattante et al., 2012 | Contribution of major amyotrophic lateral sclerosis genes to the etiology of sporadic disease | 3 |
| Lysogorskaia et al., 2015 | Genetic studies of Russian patients with amyotrophic lateral sclerosis | 3 |
| Narain et al., 2018 | Targeted next-generation sequencing reveals novel and rare variants in Indian patients with amyotrophic lateral sclerosis. | 3 |
| Soong et al., 2014 | Extensive molecular genetic survey of Taiwanese patients with amyotrophic lateral sclerosis. | 3 |
| van Blitterswijk et al., 2012 | Genetic overlap between apparently sporadic motor neuron diseases. | 3 |
| Zou et al., 2016 | The distinctive genetic architecture of ALS in mainland China | 3 |
| Amador et al., 2021 | New advances in Amyotrophic Lateral Sclerosis genetics: Towards gene therapy opportunities for familial and young cases. | 4 |
| Borg et al., 2021 | Genetic analysis of ALS cases in the isolated island population of Malta | 4 |
| Brown et al., 2012 | SOD1, ANG, TARDBP and FUS mutations in amyotrophic lateral sclerosis: a United States clinical testing lab experience. | 4 |
| Chadi et al., 2017 | Genetic analyses of patients with familial and sporadic amyotrophic lateral sclerosis in a Brazilian research center | 4 |
| Conte et al., 2012 | Classification of familial amyotrophic lateral sclerosis by family history: Effects on frequency of genes mutation | 4 |
| Cooper-Knock et al., 2017 | Targeted Genetic Screen in Amyotrophic Lateral Sclerosis Reveals Novel Genetic Variants with Synergistic Effect on Clinical Phenotype. | 4 |
| Corcia et al., 2021 | Effect of familial clustering in the genetic screening of 235 French ALS families | 4 |
| Daria et al., 2019 | Genotypes of amyotrophic lateral sclerosis in Mongolia. | 4 |
| Dekker et al., 2016 | Large-scale screening in sporadic amyotrophic lateral sclerosis identifies genetic modifiers in C9orf72 repeat carriers. | 4 |
| Edgar et al., 2021 | Mutation analysis of SOD1, C9orf72, TARDBP and FUS genes in ethnically-diverse Malaysian patients with amyotrophic lateral sclerosis (ALS). | 4 |
| Gibson et al., 2017 | The evolving genetic risk for sporadic ALS | 4 |
| Gonçalves et al., 2021 | Genetic epidemiology of familial ALS in Brazil | 4 |
| Gromicho et al., 2020 | Targeted next-generation sequencing study in familial ALS-FTD Portuguese patients negative for C9orf72 HRE | 4 |
| Krüger et al., 2016 | Rare Variants in Neurodegeneration Associated Genes Revealed by Targeted Panel Sequencing in a German ALS Cohort. | 4 |
| Lamp et al., 2018 | Twenty years of molecular analyses in amyotrophic lateral sclerosis: genetic landscape of Italian patients | 4 |
| Liu et al., 2014 | Identify mutation in amyotrophic lateral sclerosis cases using HaloPlex target enrichment system. | 4 |
| Liu et al., 2019 | Genetic spectrum and variability in Chinese patients with amyotrophic lateral sclerosis | 4 |
| Ma et al., 2022 | Genetic analysis in Chinese patients with familial or young-onset amyotrophic lateral sclerosis | 4 |
| Marjanović et al., 2017 | Comparison of the clinical and cognitive features of genetically positive ALS patients from the largest tertiary center in Serbia. | 4 |
| McCann et al., 2017 | The genotype-phenotype landscape of familial amyotrophic lateral sclerosis in Australia | 4 |
| McCluskey et al., 2014 | ALS-Plus syndrome: non-pyramidal features in a large ALS cohort. | 4 |
| Mesaros et al., 2021 | Investigating the Genetic Profile of the Amyotrophic Lateral Sclerosis/Frontotemporal Dementia (ALS-FTD) Continuum in Patients of Diverse Race, Ethnicity and Ancestry | 4 |
| Morgan et al., 2015 | Investigation of next-generation sequencing technologies as a diagnostic tool for amyotrophic lateral sclerosis | 4 |
| Müller et al., 2018 | Comprehensive analysis of the mutation spectrum in 301 German ALS families. | 4 |
| Naruse et al., 2018 | Molecular epidemiological study of familial amyotrophic lateral sclerosis in Japanese population by whole-exome sequencing and identification of novel HNRNPA1 mutation. | 4 |
| Naruse et al., 2019 | Burden of rare variants in causative genes for amyotrophic lateral sclerosis (ALS) accelerates age at onset of ALS | 4 |
| Pang et al., 2017 | Burden of rare variants in ALS genes influences survival in familial and sporadic ALS. | 4 |
| Roggenbuck et al., 2021 | Amyotrophic Lateral Sclerosis Genetic Access Program: Paving the Way for Genetic Characterization of ALS in the Clinic. | 4 |
| Scarlino et al., 2020 | Burden of rare variants in ALS and axonal hereditary neuropathy genes influence survival in ALS: Insights from a next generation sequencing study of an Italian ALS cohort | 4 |
| Sun et al., 2020 | Study on sleep-wake disorders in patients with genetic and non-genetic amyotrophic lateral sclerosis. | 4 |
| Tripolszki et al., 2017 | Genetic analysis of the SOD1 and C9ORF72 genes in Hungarian patients with amyotrophic lateral sclerosis. | 4 |
| Tripolszki et al., 2019 | Comprehensive Genetic Analysis of a Hungarian Amyotrophic Lateral Sclerosis Cohort. | 4 |
| Tsai et al., 2013 | FUS, TARDBP, and SOD1 mutations in a Taiwanese cohort with familial ALS | 4 |
| van Blitterswijk et al., 2012 | Evidence for an oligogenic basis of amyotrophic lateral sclerosis. | 4 |
| Vrabec et al., 2015 | Genetic analysis of amyotrophic lateral sclerosis in the Slovenian population. | 4 |
| Salmon et al., 2022 | The importance of offering early genetic testing in everyone with amyotrophic lateral sclerosis | 5 |

## Recommendation 4: All pALS should be offered testing with an ALS gene panel that includes *FUS*.

**GRADE rating:** A – Strong

**Strength**: This recommendation is supported by at least one study of level 1 evidence.

**Included studies**:

| **Authors and Publication Year** | **Title** | **Level of Evidence** |
| --- | --- | --- |
| Chen, 2021 | FUS mutation is probably the most common pathogenic gene for JALS, especially sporadic JALS | 1 |
| Chiò et al., 2018 | The multistep hypothesis of ALS revisited: The role of genetic mutations. | 1 |
| Grassano, et al., 2021 | Mutational Analysis of Known ALS Genes in an Italian Population-Based Cohort. | 1 |
| Wei et al., 2019 | Unique characteristics of the genetics epidemiology of amyotrophic lateral sclerosis in China. | 1 |
| Zou et al., 2017 | Genetic epidemiology of amyotrophic lateral sclerosis: a systematic review and meta-analysis. | 1 |
| Borghero et al., 2014 | Genetic architecture of ALS in Sardinia. | 2 |
| Cady et al., 2015 | Amyotrophic lateral sclerosis onset is influenced by the burden of rare variants in known amyotrophic lateral sclerosis genes. | 2 |
| Chen et al., 2020 | Clinical and genetic features of patients with amyotrophic lateral sclerosis in southern China. | 2 |
| Chen et al., 2020 | Role of genetics in amyotrophic lateral sclerosis: a large cohort study in Chinese mainland population | 2 |
| Corrado et al., 2009 | High frequency of TARDBP gene mutations in Italian patients with amyotrophic lateral sclerosis. | 2 |
| Couthouis, Julien; Raphael, Alya R.; Daneshjou, Roxana; Gitler, Aaron D. | Targeted exon capture and sequencing in sporadic amyotrophic lateral sclerosis. | 2 |
| DeJesus-Hernandez et al., 2010 | De novo truncating FUS gene mutation as a cause of sporadic amyotrophic lateral sclerosis. | 2 |
| Hou et al., 2016 | Screening of SOD1, FUS and TARDBP genes in patients with amyotrophic lateral sclerosis in central-southern China. | 2 |
| Keogh et al., 2017 | Genetic compendium of 1511 human brains available through the UK Medical Research Council Brain Banks Network Resource. | 2 |
| Kim et al., 2016 | Identification of mutations in Korean patients with amyotrophic lateral sclerosis using multigene panel testing. | 2 |
| Kwiatkowski et al., 2009 | Mutations in the FUS/TLS gene on chromosome 16 cause familial amyotrophic lateral sclerosis. | 2 |
| Lai et al., 2011 | FUS mutations in sporadic amyotrophic lateral sclerosis | 2 |
| Liu et al., 2016 | Mutation spectrum of Chinese patients with familial and sporadic amyotrophic lateral sclerosis. | 2 |
| Liu et al., 2021 | Mutation spectrum of amyotrophic lateral sclerosis in Central South China. | 2 |
| Millecamps et al., 2010 | SOD1, ANG, VAPB, TARDBP, and FUS mutations in familial amyotrophic lateral sclerosis: Genotype-phenotype correlations | 2 |
| Morgan et al., 2017 | A comprehensive analysis of rare genetic variation in amyotrophic lateral sclerosis in the UK. | 2 |
| Nakamura et al., 2016 | Next-generation sequencing of 28 ALS-related genes in a Japanese ALS cohort. | 2 |
| Nishiyama et al., 2017 | Comprehensive targeted next-generation sequencing in Japanese familial amyotrophic lateral sclerosis | 2 |
| Özoguz et al., 2015 | The distinct genetic pattern of ALS in Turkey and novel mutations | 2 |
| Pensato et al., 2020 | Sorting rare ALS genetic variants by targeted re-sequencing panel in italian patients: OPTN, VCP, and SQSTM1 variants account for 3% of rare genetic forms | 2 |
| Rademakers et al., 2010 | FUS gene mutations in familial and sporadic amyotrophic lateral sclerosis | 2 |
| Roggenbuck et al., 2020 | Incidence of pathogenic, likely pathogenic, and uncertain ALS variants in a clinic cohort | 2 |
| Rutherford et al., 2012 | Pathogenicity of exonic indels in fused in sarcoma in amyotrophic lateral sclerosis. | 2 |
| Ryan et al., 2019 | Comparison of the clinical and genetic features of amyotrophic lateral sclerosis across Cuban, Uruguayan and Irish clinic-based populations. | 2 |
| Sabatelli et al., 2013 | Mutations in the 3' untranslated region of FUS causing FUS overexpression are associated with amyotrophic lateral sclerosis. | 2 |
| Tunca et al., 2020 | Revisiting the complex architecture of ALS in Turkey: Expanding genotypes, shared phenotypes, molecular networks, and a public variant database | 2 |
| Ungaro et al., 2021 | Genetic investigation of amyotrophic lateral sclerosis patients in south Italy: a two-decade analysis | 2 |
| Waibel et al., 2013 | Truncating mutations in FUS/TLS give rise to a more aggressive ALS-phenotype than missense mutations: A clinico-genetic study in Germany | 2 |
| Yan et al., 2010 | Frameshift and novel mutations in FUS in familial amyotrophic lateral sclerosis and ALS/dementia | 2 |
| Zhang et al., 2018 | Screening for possible oligogenic pathogenesis in Chinese sporadic ALS patients. | 2 |
| Zou et al., 2012 | Screening of the FUS gene in familial and sporadic amyotrophic lateral sclerosis patients of Chinese origin. | 2 |
| Zou et al., 2021 | Novel FUS mutation Y526F causing rapidly progressive familial amyotrophic lateral sclerosis. | 2 |
| Belzil et al., 2009 | Mutations in FUS cause FALS and SALS in French and French Canadian populations. | 3 |
| Belzil et al., 2011 | Identification of novel FUS mutations in sporadic cases of amyotrophic lateral sclerosis. | 3 |
| Bertolin et al., 2014 | Improving the knowledge of amyotrophic lateral sclerosis genetics: Novel SOD1 and FUS variants | 3 |
| Chiò et al., 2012 | Extensive genetics of ALS : A population-based study in Italy | 3 |
| Drepper et al., 2011 | C-terminal FUS/TLS mutations in familial and sporadic ALS in Germany | 3 |
| Hewitt et al., 2010 | Novel FUS/TLS mutations and pathology in familial and sporadic amyotrophic lateral sclerosis. | 3 |
| Kenna et al., 2013 | Delineating the genetic heterogeneity of ALS using targeted high-throughput sequencing | 3 |
| Kwon et al., 2012 | Screening of the SOD1, FUS, TARDBP, ANG, and OPTN mutations in Korean patients with familial and sporadic ALS. | 3 |
| Narain et al., 2018 | Targeted next-generation sequencing reveals novel and rare variants in Indian patients with amyotrophic lateral sclerosis. | 3 |
| Soong et al., 2014 | Extensive molecular genetic survey of Taiwanese patients with amyotrophic lateral sclerosis. | 3 |
| Sproviero et al., 2012 | FUS mutations in sporadic amyotrophic lateral sclerosis: Clinical and genetic analysis | 3 |
| Tarlarini et al., 2015 | Novel FUS mutations identified through molecular screening in a large cohort of familial and sporadic amyotrophic lateral sclerosis. | 3 |
| Ticozzi et al., 2009 | Analysis of FUS gene mutation in familial amyotrophic lateral sclerosis within an Italian cohort. | 3 |
| van Blitterswijk et al., 2012 | Genetic overlap between apparently sporadic motor neuron diseases. | 3 |
| Vance et al., 2009 | Mutations in FUS, an RNA processing protein, cause familial amyotrophic lateral sclerosis type 6. | 3 |
| Zou et al., 2013 | De novo FUS gene mutations are associated with juvenile-onset sporadic amyotrophic lateral sclerosis in China | 3 |
| Zou et al., 2016 | The distinctive genetic architecture of ALS in mainland China | 3 |
| Akiyama et al., 2016 | Genotype-phenotype relationships in familial amyotrophic lateral sclerosis with FUS/TLS mutations in Japan | 4 |
| Amador et al., 2021 | New advances in Amyotrophic Lateral Sclerosis genetics: Towards gene therapy opportunities for familial and young cases. | 4 |
| Blair et al., 2010 | FUS mutations in amyotrophic lateral sclerosis: clinical, pathological, neurophysiological and genetic analysis. | 4 |
| Borg et al., 2021 | Genetic analysis of ALS cases in the isolated island population of Malta | 4 |
| Brown et al., 2012 | SOD1, ANG, TARDBP and FUS mutations in amyotrophic lateral sclerosis: a United States clinical testing lab experience. | 4 |
| Chadi et al., 2017 | Genetic analyses of patients with familial and sporadic amyotrophic lateral sclerosis in a Brazilian research center | 4 |
| Conte et al., 2012 | Classification of familial amyotrophic lateral sclerosis by family history: Effects on frequency of genes mutation | 4 |
| Cooper-Knock et al., 2017 | Targeted Genetic Screen in Amyotrophic Lateral Sclerosis Reveals Novel Genetic Variants with Synergistic Effect on Clinical Phenotype. | 4 |
| Corcia et al., 2021 | Effect of familial clustering in the genetic screening of 235 French ALS families | 4 |
| Damme et al., 2010 | The occurrence of mutations in FUS in a Belgian cohort of patients with familial ALS. | 4 |
| Daria et al., 2019 | Genotypes of amyotrophic lateral sclerosis in Mongolia. | 4 |
| Dekker et al., 2016 | Large-scale screening in sporadic amyotrophic lateral sclerosis identifies genetic modifiers in C9orf72 repeat carriers. | 4 |
| Edgar et al., 2021 | Mutation analysis of SOD1, C9orf72, TARDBP and FUS genes in ethnically-diverse Malaysian patients with amyotrophic lateral sclerosis (ALS). | 4 |
| Gibson et al., 2017 | The evolving genetic risk for sporadic ALS | 4 |
| Gonçalves et al., 2021 | Genetic epidemiology of familial ALS in Brazil | 4 |
| Groen et al., 2010 | FUS mutations in familial amyotrophic lateral sclerosis in the Netherlands | 4 |
| Gromicho et al., 2020 | Targeted next-generation sequencing study in familial ALS-FTD Portuguese patients negative for C9orf72 HRE | 4 |
| Hübers et al., 2015 | De novo FUS mutations are the most frequent genetic cause in early-onset German ALS patients | 4 |
| Krüger et al., 2016 | Rare Variants in Neurodegeneration Associated Genes Revealed by Targeted Panel Sequencing in a German ALS Cohort. | 4 |
| Lamp et al., 2018 | Twenty years of molecular analyses in amyotrophic lateral sclerosis: genetic landscape of Italian patients | 4 |
| Lattante et al., 2012 | Contribution of major amyotrophic lateral sclerosis genes to the etiology of sporadic disease | 3 |
| Liu et al., 2014 | Identify mutation in amyotrophic lateral sclerosis cases using HaloPlex target enrichment system. | 4 |
| Liu et al., 2019 | Genetic spectrum and variability in Chinese patients with amyotrophic lateral sclerosis | 4 |
| Ma et al., 2022 | Genetic analysis in Chinese patients with familial or young-onset amyotrophic lateral sclerosis | 4 |
| Marjanović et al., 2017 | Comparison of the clinical and cognitive features of genetically positive ALS patients from the largest tertiary center in Serbia. | 4 |
| McCann et al., 2017 | The genotype-phenotype landscape of familial amyotrophic lateral sclerosis in Australia | 4 |
| Mesaros et al., 2021 | Investigating the Genetic Profile of the Amyotrophic Lateral Sclerosis/Frontotemporal Dementia (ALS-FTD) Continuum in Patients of Diverse Race, Ethnicity and Ancestry | 4 |
| Morgan et al., 2015 | Investigation of next-generation sequencing technologies as a diagnostic tool for amyotrophic lateral sclerosis | 4 |
| Müller et al., 2018 | Comprehensive analysis of the mutation spectrum in 301 German ALS families. | 4 |
| Naruse et al., 2018 | Molecular epidemiological study of familial amyotrophic lateral sclerosis in Japanese population by whole-exome sequencing and identification of novel HNRNPA1 mutation. | 4 |
| Naruse et al., 2019 | Burden of rare variants in causative genes for amyotrophic lateral sclerosis (ALS) accelerates age at onset of ALS | 4 |
| Pang et al., 2017 | Burden of rare variants in ALS genes influences survival in familial and sporadic ALS. | 4 |
| Roggenbuck et al., 2021 | Amyotrophic Lateral Sclerosis Genetic Access Program: Paving the Way for Genetic Characterization of ALS in the Clinic. | 4 |
| Scarlino et al., 2020 | Burden of rare variants in ALS and axonal hereditary neuropathy genes influence survival in ALS: Insights from a next generation sequencing study of an Italian ALS cohort | 4 |
| Sun et al., 2020 | Study on sleep-wake disorders in patients with genetic and non-genetic amyotrophic lateral sclerosis. | 4 |
| Syriani et al., 2011 | FUS/TLS gene mutations are the second most frequent cause of familial ALS in the Spanish population | 4 |
| Tripolszki et al., 2019 | Comprehensive Genetic Analysis of a Hungarian Amyotrophic Lateral Sclerosis Cohort. | 4 |
| Tsai et al., 2013 | FUS, TARDBP, and SOD1 mutations in a Taiwanese cohort with familial ALS | 4 |
| van Blitterswijk et al., 2012 | Evidence for an oligogenic basis of amyotrophic lateral sclerosis. | 4 |
| Van Langenhove et al., 2010 | Genetic contribution of FUS to frontotemporal lobar degeneration. | 4 |
| Vrabec et al., 2015 | Genetic analysis of amyotrophic lateral sclerosis in the Slovenian population. | 4 |
| Waibel et al., 2010 | Novel missense and truncating mutations in FUS/TLS in familial ALS. | 4 |
| Zou et al., 2016 | Mutations in FUS are the most frequent genetic cause in juvenile sporadic ALS patients of Chinese origin | 4 |
| Salmon et al., 2022 | The importance of offering early genetic testing in everyone with amyotrophic lateral sclerosis | 5 |

**Recommendation 5: All pALS should be offered testing with an ALS gene panel that includes *TARDBP*.**

**GRADE rating:** A - Strong

**Strength**: This recommendation is supported by at least one study of level 1 evidence.

**Included studies**:

| **Authors and Publication Year** | **Title** | **Level of Evidence** |
| --- | --- | --- |
| Chiò et al., 2018 | The multistep hypothesis of ALS revisited: The role of genetic mutations. | 1 |
| Grassano, et al., 2021 | Mutational Analysis of Known ALS Genes in an Italian Population-Based Cohort. | 1 |
| Wei et al., 2019 | Unique characteristics of the genetics epidemiology of amyotrophic lateral sclerosis in China. | 1 |
| Zou et al., 2017 | Genetic epidemiology of amyotrophic lateral sclerosis: a systematic review and meta-analysis. | 1 |
| Borghero et al., 2014 | Genetic architecture of ALS in Sardinia. | 2 |
| Cady et al., 2015 | Amyotrophic lateral sclerosis onset is influenced by the burden of rare variants in known amyotrophic lateral sclerosis genes. | 2 |
| Chen et al., 2020 | Role of genetics in amyotrophic lateral sclerosis: a large cohort study in Chinese mainland population | 2 |
| Chen et al., 2020 | Clinical and genetic features of patients with amyotrophic lateral sclerosis in southern China. | 2 |
| Chen et al., 2021 | Novel TARDBP missense mutation caused familial amyotrophic lateral sclerosis with frontotemporal dementia and parkinsonism. | 2 |
| Corrado et al., 2009 | High frequency of TARDBP gene mutations in Italian patients with amyotrophic lateral sclerosis. | 2 |
| Couthouis et al., 2014 | Targeted exon capture and sequencing in sporadic amyotrophic lateral sclerosis. | 2 |
| DeJesus-Hernandez et al., 2010 | De novo truncating FUS gene mutation as a cause of sporadic amyotrophic lateral sclerosis. | 2 |
| Erazo et al., 2022 | Epidemiological and genetic features of amyotrophic lateral sclerosis in Latin America and the Caribbean: a systematic review | 2 |
| Feng et al., 2021 | Genetic and clinical features of Chinese sporadic amyotrophic lateral sclerosis patients with TARDBP mutations | 2 |
| Hou et al., 2016 | Screening of SOD1, FUS and TARDBP genes in patients with amyotrophic lateral sclerosis in central-southern China. | 2 |
| Ju et al., 2016 | Two distinct clinical features and cognitive impairment in amyotrophic lateral sclerosis patients with TARDBP gene mutations in the Chinese population. | 2 |
| Kim et al., 2016 | Identification of mutations in Korean patients with amyotrophic lateral sclerosis using multigene panel testing. | 2 |
| Liu et al., 2016 | Mutation spectrum of Chinese patients with familial and sporadic amyotrophic lateral sclerosis. | 2 |
| Liu et al., 2021 | Mutation spectrum of amyotrophic lateral sclerosis in Central South China. | 2 |
| Millecamps et al., 2010 | SOD1, ANG, VAPB, TARDBP, and FUS mutations in familial amyotrophic lateral sclerosis: Genotype-phenotype correlations | 2 |
| Morgan et al., 2017 | A comprehensive analysis of rare genetic variation in amyotrophic lateral sclerosis in the UK. | 2 |
| Nakamura et al., 2016 | Next-generation sequencing of 28 ALS-related genes in a Japanese ALS cohort. | 2 |
| Nishiyama et al., 2017 | Comprehensive targeted next-generation sequencing in Japanese familial amyotrophic lateral sclerosis | 2 |
| Özoguz et al., 2015 | The distinct genetic pattern of ALS in Turkey and novel mutations | 2 |
| Pensato et al., 2020 | Sorting rare ALS genetic variants by targeted re-sequencing panel in italian patients: OPTN, VCP, and SQSTM1 variants account for 3% of rare genetic forms | 2 |
| Roggenbuck et al., 2020 | Incidence of pathogenic, likely pathogenic, and uncertain ALS variants in a clinic cohort | 2 |
| Rutherford et al., 2008 | Novel mutations in TARDBP(TDP-43) in patients with familial amyotrophic lateral sclerosis | 2 |
| Ryan et al., 2019 | Comparison of the clinical and genetic features of amyotrophic lateral sclerosis across Cuban, Uruguayan and Irish clinic-based populations. | 2 |
| Ticozzi et al., 2011 | Mutational analysis of TARDBP in neurodegenerative diseases. | 2 |
| Tunca et al., 2020 | Revisiting the complex architecture of ALS in Turkey: Expanding genotypes, shared phenotypes, molecular networks, and a public variant database | 2 |
| Ungaro et al., 2021 | Genetic investigation of amyotrophic lateral sclerosis patients in south Italy: a two-decade analysis | 2 |
| Van Deerlin et al., 2008 | TARDBP mutations in amyotrophic lateral sclerosis with TDP-43 neuropathology: a genetic and histopathological analysis | 2 |
| Wang et al., 2020 | Identification of novel FUS and TARDBP gene mutations in Chinese amyotrophic lateral sclerosis patients with HRM analysis. | 2 |
| Zhang et al., 2018 | Screening for possible oligogenic pathogenesis in Chinese sporadic ALS patients. | 2 |
| Bertolin et al., 2014 | Improving the knowledge of amyotrophic lateral sclerosis genetics: Novel SOD1 and FUS variants | 3 |
| Chiò et al., 2011 | Large proportion of amyotrophic lateral sclerosis cases in sardinia due to a single founder mutation of the TARDBP gene | 3 |
| Chiò et al., 2012 | Extensive genetics of ALS : A population-based study in Italy | 3 |
| Conforti et al., 2011 | TARDBP gene mutations in south Italian patients with amyotrophic lateral sclerosis. | 3 |
| Czell et al., 2013 | Phenotypes in Swiss patients with familial ALS carrying TARDBP mutations | 3 |
| Del Bo et al., 2009. | TARDBP (TDP-43) sequence analysis in patients with familial and sporadic ALS: identification of two novel mutations. | 3 |
| Gijselinck et al., 2009 | Neuronal inclusion protein TDP-43 has no primary genetic role in FTD and ALS | 3 |
| Huang et al., 2012 | TARDBP gene mutations among Chinese patients with sporadic amyotrophic lateral sclerosis | 3 |
| Kamada et al., 2009 | Screening for TARDBP mutations in Japanese familial amyotrophic lateral sclerosis. | 3 |
| Kenna et al., 2013 | Delineating the genetic heterogeneity of ALS using targeted high-throughput sequencing | 3 |
| Kirby et al., 2010 | Broad clinical phenotypes associated with TAR-DNA binding protein (TARDBP) mutations in amyotrophic lateral sclerosis. | 3 |
| Kwon et al., 2012 | Screening of the SOD1, FUS, TARDBP, ANG, and OPTN mutations in Korean patients with familial and sporadic ALS. | 3 |
| Lattante et al., 2012 | Contribution of major amyotrophic lateral sclerosis genes to the etiology of sporadic disease | 3 |
| Lysogorskaia et al., 2015 | Genetic studies of Russian patients with amyotrophic lateral sclerosis | 3 |
| Narain et al., 2018 | Targeted next-generation sequencing reveals novel and rare variants in Indian patients with amyotrophic lateral sclerosis. | 3 |
| Soong et al., 2014 | Extensive molecular genetic survey of Taiwanese patients with amyotrophic lateral sclerosis. | 3 |
| van Blitterswijk et al., 2012 | Genetic overlap between apparently sporadic motor neuron diseases. | 3 |
| Williams et al., 2009 | A novel TARDBP mutation in an Australian amyotrophic lateral sclerosis kindred. | 3 |
| Ye et al., 2013 | Absence of Mutations in Exon 6 of the TARDBP Gene in 207 Chinese Patients with Sporadic Amyotrophic Lateral Sclerosis | 3 |
| Zou et al., 2016 | The distinctive genetic architecture of ALS in mainland China | 3 |
| Amador et al., 2021 | New advances in Amyotrophic Lateral Sclerosis genetics: Towards gene therapy opportunities for familial and young cases. | 4 |
| Borg et al., 2021 | Genetic analysis of ALS cases in the isolated island population of Malta | 4 |
| Brown et al., 2012 | SOD1, ANG, TARDBP and FUS mutations in amyotrophic lateral sclerosis: a United States clinical testing lab experience. | 4 |
| Chadi et al., 2017 | Genetic analyses of patients with familial and sporadic amyotrophic lateral sclerosis in a Brazilian research center | 4 |
| Conte et al., 2012 | Classification of familial amyotrophic lateral sclerosis by family history: Effects on frequency of genes mutation | 4 |
| Cooper-Knock et al., 2017 | Targeted Genetic Screen in Amyotrophic Lateral Sclerosis Reveals Novel Genetic Variants with Synergistic Effect on Clinical Phenotype. | 4 |
| Corcia et al., 2021 | Effect of familial clustering in the genetic screening of 235 French ALS families | 4 |
| Dekker et al., 2016 | Large-scale screening in sporadic amyotrophic lateral sclerosis identifies genetic modifiers in C9orf72 repeat carriers. | 4 |
| Edgar et al., 2021 | Mutation analysis of SOD1, C9orf72, TARDBP and FUS genes in ethnically-diverse Malaysian patients with amyotrophic lateral sclerosis (ALS). | 4 |
| Gibson et al., 2017 | The evolving genetic risk for sporadic ALS | 4 |
| Gonçalves et al., 2021 | Genetic epidemiology of familial ALS in Brazil | 4 |
| Gromicho et al., 2020 | Targeted next-generation sequencing study in familial ALS-FTD Portuguese patients negative for C9orf72 HRE | 4 |
| Krüger et al., 2016 | Rare Variants in Neurodegeneration Associated Genes Revealed by Targeted Panel Sequencing in a German ALS Cohort. | 4 |
| Lamp et al., 2018 | Twenty years of molecular analyses in amyotrophic lateral sclerosis: genetic landscape of Italian patients | 4 |
| Liu et al., 2014 | Identify mutation in amyotrophic lateral sclerosis cases using HaloPlex target enrichment system. | 4 |
| Liu et al., 2019 | Genetic spectrum and variability in Chinese patients with amyotrophic lateral sclerosis | 4 |
| Luquin et al., 2009 | Genetic variants in the promoter of TARDBP in sporadic amyotrophic lateral sclerosis. | 4 |
| Marjanović et al., 2017 | Comparison of the clinical and cognitive features of genetically positive ALS patients from the largest tertiary center in Serbia. | 4 |
| McCann et al., 2017 | The genotype-phenotype landscape of familial amyotrophic lateral sclerosis in Australia | 4 |
| McCluskey et al., 2014 | ALS-Plus syndrome: non-pyramidal features in a large ALS cohort. | 4 |
| Mentula et al., 2012 | TARDBP mutations are not a frequent cause of ALS in Finnish patients | 4 |
| Mesaros et al., 2021 | Investigating the Genetic Profile of the Amyotrophic Lateral Sclerosis/Frontotemporal Dementia (ALS-FTD) Continuum in Patients of Diverse Race, Ethnicity and Ancestry | 4 |
| Morgan et al., 2015 | Investigation of next-generation sequencing technologies as a diagnostic tool for amyotrophic lateral sclerosis | 4 |
| Müller et al., 2018 | Comprehensive analysis of the mutation spectrum in 301 German ALS families. | 4 |
| Naruse et al., 2018 | Molecular epidemiological study of familial amyotrophic lateral sclerosis in Japanese population by whole-exome sequencing and identification of novel HNRNPA1 mutation. | 4 |
| Naruse et al., 2019 | Burden of rare variants in causative genes for amyotrophic lateral sclerosis (ALS) accelerates age at onset of ALS | 4 |
| Origone et al., 2010 | Enlarging clinical spectrum of FALS with TARDBP gene mutations: S393L variant in an Italian family showing phenotypic variability and relevance for genetic counseling. | 4 |
| Orrù et al., 2012 | High frequency of the TARDBP p.Ala382Thr mutation in Sardinian patients with amyotrophic lateral sclerosis. | 4 |
| Pang et al., 2017 | Burden of rare variants in ALS genes influences survival in familial and sporadic ALS. | 4 |
| Piaceri et al., 2012 | Clinical heterogeneity in Italian patients with amyotrophic lateral sclerosis. | 4 |
| Roggenbuck et al., 2021 | Amyotrophic Lateral Sclerosis Genetic Access Program: Paving the Way for Genetic Characterization of ALS in the Clinic. | 4 |
| Scarlino et al., 2020 | Burden of rare variants in ALS and axonal hereditary neuropathy genes influence survival in ALS: Insights from a next generation sequencing study of an Italian ALS cohort | 4 |
| Solski et al., 2012 | A novel TARDBP insertion/deletion mutation in the flail arm variant of amyotrophic lateral sclerosis | 4 |
| Sun et al., 2020 | Study on sleep-wake disorders in patients with genetic and non-genetic amyotrophic lateral sclerosis. | 4 |
| Tripolszki et al., 2019 | Comprehensive Genetic Analysis of a Hungarian Amyotrophic Lateral Sclerosis Cohort. | 4 |
| Tsai et al., 2013 | FUS, TARDBP, and SOD1 mutations in a Taiwanese cohort with familial ALS | 4 |
| van Blitterswijk et al., 2012 | Evidence for an oligogenic basis of amyotrophic lateral sclerosis. | 4 |
| Vrabec et al., 2015 | Genetic analysis of amyotrophic lateral sclerosis in the Slovenian population. | 4 |
| Xiong et al., 2010 | Association between novel TARDBP mutations and Chinese patients with amyotrophic lateral sclerosis. | 4 |
| Xu et al., 2018 | High frequency of the TARDBP p.M337Â V mutation among south-eastern Chinese patients with familial amyotrophic lateral sclerosis. | 4 |
| Zou et al., 2012 | Screening of the TARDBP gene in familial and sporadic amyotrophic lateral sclerosis patients of Chinese origin. | 4 |
| Salmon et al., 2022 | The importance of offering early genetic testing in everyone with amyotrophic lateral sclerosis | 5 |

## Recommendation 6: Additional genetic testing should include genes strongly and definitively associated with ALS as determined by [ClinGen](https://clinicalgenome.org/)

**GRADE rating:** D - Expert

**Strength:** This recommendation is supported by Expert Opinion

**Recommendation 7: In the event of an FDA–approved gene-targeted therapy, all pALS should be offered testing for that gene.**

**GRADE rating:** D - Expert

**Strength:** This recommendation is supported by Expert Opinion

Genetic Counseling Recommendations

## 8: Genetic counseling and education should be provided to all pALS.

**GRADE rating:** C - Weak

**Strength**: This recommendation is supported by at least one study of level 3 evidence.

**Included Studies**:

| **Authors and Publication Year** | **Title** | **Level of Evidence** |
| --- | --- | --- |
| Al-Chalabi, 2010 | An estimate of amyotrophic lateral sclerosis heritability using twin data. | 3 |
| Chio et al, 2018 | The multistep hypothesis of ALS revisited: The role of genetic mutations. | 3 |
| Klepek et al, 2019 | Lack of consensus in ALS genetic testing practices and divergent views between ALS clinicians and patients | 3 |
| Salman et al, 2021 | Genetic testing for amyotrophic lateral sclerosis in Canada-an assessment of current practices | 3 |
| Wagner et al, 2018 | Patients with sporadic and familial amyotrophic lateral sclerosis found value in genetic testing | 3 |
| Wagner et al, 2017 | Patients with Amyotrophic Lateral Sclerosis Have High Interest in and Limited Access to Genetic Testing | 3 |
| Crook et al., 2022 | Patient and Relative Experiences and Decision-making About  Genetic Testing and Counseling for Familial ALS and FTD | 3 |
| Al-Chalabi, 2017 | Gene discovery in amyotrophic lateral sclerosis: implications for clinical management. | 5 |
| Anderson, 2011 | Clinical genetics of amyotrophic lateral sclerosis: What do we really know? | 5 |
| Chio el al, 2014 | Genetic counseling in ALS: facts, uncertainties and clinical suggestions. | 5 |
| Dharmadasa et al, 2022 | Genetic testing in motor neurone disease | 5 |
| Fong et al, 2012 | Genetic counseling for ftd/als caused by the c9orf72 hexanucleotide expansion | 5 |
| Roggenbuck et al, 2017 | Genetic testing and genetic counseling for amyotrophic lateral sclerosis: an update for clinicians. | 5 |
| Roggenbuck et al, 2020 | Genetic Testing for Amyotrophic Lateral Sclerosis and Frontotemporal Dementia: Impact on Clinical Management. | 5 |
| Shtilbans et al, 2020 | Lifetime Risk and Heritability of Amyotrophic Lateral Sclerosis. | 5 |
| Talbot, 2017 | Should all patients with ALS have genetic testing? | 5 |
| Traynor | A roadmap for genetic testing in ALS | 5 |
| Turner et al, 2017 | Genetic screening in sporadic ALS and FTD | 5 |
| Volk et al, 2018 | Current knowledge and recent insights into the genetic basis of amyotrophic lateral sclerosis. | 5 |
| Salmon et al., 2022 | The importance of offering early genetic testing in everyone with amyotrophic lateral sclerosis | 5 |

## Recommendation 9: Genetic counseling should precede the offer of testing.

**GRADE rating:** C - Weak

**Strength**: This recommendation is supported by at least one study of level 3 evidence.

**Included Studies**:

| **Authors and Publication Year** | **Title** | **Level of Evidence** |
| --- | --- | --- |
| Benatar et al, 2016 | Presymptomatic ALS genetic counseling and testing | 3 |
| Crook et al 2017 | The C9orf72 hexanucleotide repeat expansion presents a challenge for testing laboratories and genetic counseling. | 3 |
| Crook et al., 2022 | Patient and Relative Experiences and Decision-making About  Genetic Testing and Counseling for Familial ALS and FTD | 3 |
| Wagner et al, 2018 | Patients with Amyotrophic Lateral Sclerosis Have High Interest in and Limited Access to Genetic Testing | 3 |
| Anderson, 2011 | Clinical genetics of amyotrophic lateral sclerosis: What do we really know? | 5 |
| Chio el al, 2014 | Genetic counseling in ALS: facts, uncertainties and clinical suggestions. | 5 |
| Dharmadasa et al, 2022 | Genetic testing in motor neurone disease | 5 |
| Eisen et al, 2013 | Ethical considerations in the management of amyotrophic lateral sclerosis. | 5 |
| Fong et al, 2012 | Genetic counseling for ftd/als caused by the c9orf72 hexanucleotide expansion | 5 |
| Hogden et al 2017 | Patient-centered decision making in amyotrophic lateral sclerosis: where are we? | 5 |
| Roggenbuck et al, 2017 | Genetic testing and genetic counseling for amyotrophic lateral sclerosis: an update for clinicians. | 5 |
| Roggenbuck et al, 2020 | Genetic Testing for Amyotrophic Lateral Sclerosis and Frontotemporal Dementia: Impact on Clinical Management. | 5 |
| Turner et al, 2017 | Genetic screening in sporadic ALS and FTD | 5 |
| Volk et al, 2018 | Current knowledge and recent insights into the genetic basis of amyotrophic lateral sclerosis. | 5 |

## Recommendation 10: A pedigree going back 3-generations at minimum should be documented.

**GRADE rating:** B - Moderate

**Strength of recommendation**: This recommendation is supported by at least one study of level 2 evidence.

**Included Studies**:

| **Authors and Publication Year** | **Title** | **Level of Evidence** |
| --- | --- | --- |
| Byrne et al, 2011 | Rate of familial amyotrophic lateral sclerosis: a systematic review and meta-analysis. | 2 |
| Anderson, 2000 | Genetic factors in the early diagnosis of ALS. | 5 |
| Anderson, 2011 | Clinical genetics of amyotrophic lateral sclerosis: What do we really know? | 5 |
| Dharmadasa et al, 2022 | Genetic testing in motor neurone disease | 5 |
| Fong et al, 2012 | Genetic counseling for ftd/als caused by the c9orf72 hexanucleotide expansion | 5 |
| Roggenbuck et al, 2017 | Genetic testing and genetic counseling for amyotrophic lateral sclerosis: an update for clinicians. | 5 |
| Roggenbuck et al, 2020 | Genetic Testing for Amyotrophic Lateral Sclerosis and Frontotemporal Dementia: Impact on Clinical Management. | 5 |

Recommendation 11: **The pedigree should ascertain ALS and related motor neuron disorders (e.g. PLS, PMA, PBP), frontotemporal dementia, other dementias, movement disorders, and psychiatric disease.**

**GRADE rating:** B - Moderate

**Strength**: This recommendation is supported by at least one study of level 2 evidence.

**Included Studies**:

| **Authors and Publication Year** | **Title** | **Level of Evidence** |
| --- | --- | --- |
| Byrne et al, 2011 | Rate of familial amyotrophic lateral sclerosis: a systematic review and meta-analysis. | 2 |
| Kaivola et a, 2019 | C9orf72 hexanucleotide repeat length in older population: normal variation and effects on cognition. | 3 |
| Roggenbuck et al, 2020 | Incidence of pathogenic, likely pathogenic, and uncertain ALS variants in a clinic cohort | 3 |
| Roggenbuck et al, 2021 | Amyotrophic Lateral Sclerosis Genetic Access Program: Paving the Way for Genetic Characterization of ALS in the Clinic. | 3 |
| van Blitterswijk et al, 2012 | Genetic overlap between apparently sporadic motor neuron diseases. | 3 |
| Dharmadasa et al, 2022 | Genetic testing in motor neurone disease | 5 |
| Esselin et al, 2020 | Clinical Phenotype and Inheritance in Patients With C9ORF72 Hexanucleotide Repeat Expansion: Results From a Large French Cohort | 5 |
| Fong et al, 2012 | Genetic counseling for ftd/als caused by the c9orf72 hexanucleotide expansion | 5 |
| Roggenbuck et al, 2017 | Genetic testing and genetic counseling for amyotrophic lateral sclerosis: an update for clinicians. | 5 |
| Roggenbuck et al, 2020 | Genetic Testing for Amyotrophic Lateral Sclerosis and Frontotemporal Dementia: Impact on Clinical Management. | 5 |
| Shu et al, 2016 | The Association between C9orf72 Repeats and Risk of Alzheimer's Disease and Amyotrophic Lateral Sclerosis: A Meta-Analysis | 2 |
| Turner et al, 2017 | Genetic screening in sporadic ALS and FTD | 5 |
| van der Ende et al, 2021 | Unraveling the clinical spectrum and the role of repeat length in C9ORF72 repeat expansions. | 5 |

## Recommendation 12: Genetic counseling should include personalized risk assessments for the likelihood of a genetic etiology, and the likelihood of positive results on testing of currently known genes.

**GRADE rating:** B - Moderate , B - Moderate

**Strength**: This recommendation is supported with at least one study of level 2 evidence.

**Included Studies**:

| **Authors and Publication Year** | **Title** | **Level of Evidence** |
| --- | --- | --- |
| Byrne et al, 2011 | Rate of familial amyotrophic lateral sclerosis: a systematic review and meta-analysis. | 2 |
| Amador et al, 2021 | New advances in Amyotrophic Lateral Sclerosis genetics: Towards gene therapy opportunities for familial and young cases. | 3 |
| Conte et al, 2012 | Classification of familial amyotrophic lateral sclerosis by family history: Effects on frequency of genes mutation | 3 |
| Corcia et al, 2021 | Effect of familial clustering in the genetic screening of 235 French ALS families | 3 |
| Curtis et al, 2017 | Sex differences in the prevalence of genetic mutations in FTD and ALS | 2 |
| Mehta et al, 2018 | Younger age of onset in familial amyotrophic lateral sclerosis is a result of pathogenic gene variants, rather than ascertainment bias | 3 |
| Mesaros et al, 2022 | Investigating the Genetic Profile of the Amyotrophic Lateral Sclerosis/Frontotemporal Dementia (ALS-FTD) Continuum in Patients of Diverse Race, Ethnicity and Ancestry | 3 |
| Quansah et al, 2015 | Motor Neuron Diseases in Sub-Saharan Africa: The Need for More Population-Based Studies. | 3 |
| Roggenbuck et al, 2021 | Amyotrophic Lateral Sclerosis Genetic Access Program: Paving the Way for Genetic Characterization of ALS in the Clinic. | 3 |
| Vajda et al, 2017 | Genetic testing in ALS. | 3 |
| Anderson, 2011 | Clinical genetics of amyotrophic lateral sclerosis: What do we really know? | 5 |
| Dharmadasa et al, 2022 | Genetic testing in motor neurone disease | 5 |
| Fong et al, 2012 | Genetic counseling for ftd/als caused by the c9orf72 hexanucleotide expansion | 5 |
| Roggenbuck et al, 2017 | Genetic testing and genetic counseling for amyotrophic lateral sclerosis: an update for clinicians. | 5 |
| Shepheard et al, 2021 | Value of systematic genetic screening of patients with amyotrophic lateral sclerosis | 3 |
| Shtilbans et al, 2020 | Lifetime Risk and Heritability of Amyotrophic Lateral Sclerosis. | 5 |

## Recommendation 13: Genetic counseling should include discussion of genetic heterogeneity.

**GRADE rating:** B - Moderate

**Strength**: This recommendation is supported by at least one study of Level 2 evidence.

**Included Studies**:

| **Authors and Publication Year** | **Title** | **Level of Evidence** |
| --- | --- | --- |
| Byrne et al, 2011 | Rate of familial amyotrophic lateral sclerosis: a systematic review and meta-analysis. | 2 |
| Shu et al, 2016 | The Association between C9orf72 Repeats and Risk of Alzheimer's Disease and Amyotrophic Lateral Sclerosis: A Meta-Analysis | 2 |
| Chio et al, 2018 | The multistep hypothesis of ALS revisited: The role of genetic mutations. | 3 |
| Vajda et al, 2017 | Genetic testing in ALS. | 3 |
| Vucic et al, 2020 | ALS is a multistep process in South Korean, Japanese, and Australian patients. | 3 |
| Anderson, 2011 | Clinical genetics of amyotrophic lateral sclerosis: What do we really know? | 5 |
| Byrne et al, | Proposed criteria for familial amyotrophic lateral sclerosis. | 5 |
| Chio el al, 2014 | Genetic counseling in ALS: facts, uncertainties and clinical suggestions. | 5 |
| Dharmadasa et al, 2022 | Genetic testing in motor neurone disease | 5 |
| Fong et al, 2012 | Genetic counseling for ftd/als caused by the c9orf72 hexanucleotide expansion | 5 |
| Roggenbuck et al, 2017 | Genetic testing and genetic counseling for amyotrophic lateral sclerosis: an update for clinicians. | 5 |
| Shtilbans et al, 2020 | Lifetime Risk and Heritability of Amyotrophic Lateral Sclerosis. | 5 |
| Su et al, 2014 | Genetic heterogeneity of amyotrophic lateral sclerosis: Implications for clinical practice and research. | 5 |
| Volk et al, 2018 | Current knowledge and recent insights into the genetic basis of amyotrophic lateral sclerosis. | 5 |

## Recommendation 14: Genetic counseling should include a discussion of inheritance patterns.

**GRADE rating:** B - Moderate

**Strength of recommendation**: This recommendation is supported with at least one study of level 2 evidence.

**Included Studies**:

| **Authors and Publication Year** | **Title** | **Level of Evidence** |
| --- | --- | --- |
| Byrne et al, 2011 | Rate of familial amyotrophic lateral sclerosis: a systematic review and meta-analysis. | 2 |
| Bradley et al, 2005 | Patterns of inheritance in familial ALS. | 3 |
| Ross et al, 2020 | Oligogenicity, C9orf72 expansion, and variant severity in ALS | 3 |
| Andersen, 2011 | Clinical genetics of amyotrophic lateral sclerosis: What do we really know? | 5 |
| Byrne et al, | Proposed criteria for familial amyotrophic lateral sclerosis. | 5 |
| Chio el al, 2014 | Genetic counseling in ALS: facts, uncertainties and clinical suggestions. | 5 |
| Dharmadasa et al, 2022 | Genetic testing in motor neurone disease | 5 |
| Fong et al, 2012 | Genetic counseling for ftd/als caused by the c9orf72 hexanucleotide expansion | 5 |
| Roggenbuck et al, 2017 | Genetic testing and genetic counseling for amyotrophic lateral sclerosis: an update for clinicians. | 5 |
| Volk et al, 2018 | Current knowledge and recent insights into the genetic basis of amyotrophic lateral sclerosis. | 5 |

## Recommendation 15: Genetic counseling should include a discussion of penetrance.

**GRADE rating:** C – Weak

**Strength of recommendation**: This recommendation is supported with at least one study of level 3 evidence.

**Included Studies**:

| **Authors and Publication Year** | **Title** | **Level of Evidence** |
| --- | --- | --- |
| Crook et al., 2019 | The C9orf72 hexanucleotide repeat expansion presents a challenge for testing laboratories and genetic counseling. | 3 |
| Crook et al., 2022 | Patient and Relative Experiences and Decision-making About  Genetic Testing and Counseling for Familial ALS and FTD | 3 |
| Fanos et al, 2004 | You have shown me my end": attitudes toward presymptomatic testing for familial amyotrophic lateral sclerosis." | 3 |
| Fournier et al, 2019 | Relations between C9orf72 expansion size in blood, age at onset, age at collection and transmission across generations in patients and presymptomatic carriers | 3 |
| Majounie et al, 2012 | Frequency of the C9orf72 hexanucleotide repeat expansion in patients with amyotrophic lateral sclerosis and frontotemporal dementia: A cross-sectional study | 3 |
| Murphy et al, 2017 | Age-related penetrance of the C9orf72 repeat expansion. | 3 |
| Ross et al, 2020 | Oligogenicity, C9orf72 expansion, and variant severity in ALS | 3 |
| Rutherford et al, 2012 | Length of normal alleles of C9ORF72 GGGGCC repeat do not influence disease phenotype | 3 |
| Vajda et al, 2017 | Genetic testing in ALS. | 3 |
| van Mossevelde et al, 2017 | Clinical Evidence of Disease Anticipation in Families Segregating a C9orf72 Repeat Expansion. | 3 |
| Anderson, 2011 | Clinical genetics of amyotrophic lateral sclerosis: What do we really know? | 5 |
| Byrne et al, 2011 | Proposed criteria for familial amyotrophic lateral sclerosis. | 5 |
| Chio el al, 2014 | Genetic counseling in ALS: facts, uncertainties and clinical suggestions. | 5 |
| Crook et al , 2017 | Predictive genetic testing for amyotrophic lateral sclerosis and frontotemporal dementia: genetic counseling considerations. | 5 |
| Dharmadasa et al, 2022 | Genetic testing in motor neurone disease | 5 |
| Fong et al, 2012 | Genetic counseling for ftd/als caused by the c9orf72 hexanucleotide expansion | 5 |
| Roggenbuck et al, 2017 | Genetic testing and genetic counseling for amyotrophic lateral sclerosis: an update for clinicians. | 5 |
| Turner et al, 2017 | Genetic screening in sporadic ALS and FTD | 5 |
| Volk et al, 2018 | Current knowledge and recent insights into the genetic basis of amyotrophic lateral sclerosis. | 5 |

## Recommendation 16: Genetic counseling should include personalized risk assessment for recurrence in relatives.

**GRADE rating:** B - Moderate

**Strength of recommendation**: This recommendation is supported with at least one study of level 2 evidence.

**Included Studies**:

| **Authors and Publication Year** | **Title** | **Level of Evidence** |
| --- | --- | --- |
| Gibson et al, 2014 | Familial clustering of ALS in a population-based resource. | 2 |
| Hanby et al, 2011 | The risk to relatives of patients with sporadic amyotrophic lateral sclerosis. | 3 |
| Pamphlett et al, 2012 | Transmission of C9orf72 hexanucleotide repeat expansions in sporadic amyotrophic lateral sclerosis: An Australian trio study | 3 |
| Anderson, 2011 | Clinical genetics of amyotrophic lateral sclerosis: What do we really know? | 5 |
| Byrne et al, | Proposed criteria for familial amyotrophic lateral sclerosis. | 5 |
| Fong et al, 2012 | Genetic counseling for ftd/als caused by the c9orf72 hexanucleotide expansion | 5 |
| Lee et al, 2017 | Genetic testing and reproductive choice in neurological disorders. | 5 |
| Roggenbuck et al, 2017 | Genetic testing and genetic counseling for amyotrophic lateral sclerosis: an update for clinicians. | 5 |
| Shtilbans et al, 2020 | Lifetime Risk and Heritability of Amyotrophic Lateral Sclerosis. | 5 |
| Turner et al, 2017 | Genetic screening in sporadic ALS and FTD | 5 |
| Volk et al, 2018 | Current knowledge and recent insights into the genetic basis of amyotrophic lateral sclerosis. | 5 |

## 17: Pretest counseling should prepare individuals for possible personal, psychological, and economic impacts of testing on themselves and their family members.

**GRADE rating:** C - Weak

**Strength**: This recommendation is supported with at least one study of level 3 evidence.

**Included Studies**:

| **Authors and Publication Year** | **Title** | **Level of Evidence** |
| --- | --- | --- |
| Crook et al., 2022 | Patient and Relative Experiences and Decision-making About  Genetic Testing and Counseling for Familial ALS and FTD | 3 |
| Benatar et al, 2016 | Presymptomatic ALS genetic counseling and testing | 3 |
| Fanos et al, 2004 | You have shown me my end": attitudes toward presymptomatic testing for familial amyotrophic lateral sclerosis." | 3 |
| Fanos et al, 2011 | Impact of presymptomatic genetic testing for familial amyotrophic lateral sclerosis | 3 |
| Chio el al, 2014 | Genetic counseling in ALS: facts, uncertainties and clinical suggestions. | 5 |
| Crook et al , 2019 | Predictive genetic testing for amyotrophic lateral sclerosis and frontotemporal dementia: genetic counseling considerations. | 5 |
| Dharmadasa et al, 2022 | Genetic testing in motor neurone disease | 5 |
| Eisen et al, 2013 | Ethical considerations in the management of amyotrophic lateral sclerosis. | 5 |
| Fong et al, 2012 | Genetic counseling for ftd/als caused by the c9orf72 hexanucleotide expansion | 5 |
| Hogden et al 2017 | Patient-centered decision making in amyotrophic lateral sclerosis: where are we? | 5 |
| Lee et al, 2017 | Genetic testing and reproductive choice in neurological disorders. | 5 |
| Roggenbuck et al 2020 | Genetic Testing for Amyotrophic Lateral Sclerosis and Frontotemporal Dementia: Impact on Clinical Management. | 5 |
| Roggenbuck et al, 2017 | Genetic testing and genetic counseling for amyotrophic lateral sclerosis: an update for clinicians. | 5 |
| Volk et al, 2018 | Current knowledge and recent insights into the genetic basis of amyotrophic lateral sclerosis. | 5 |

**Recommendation 18: pALS should be informed of the range of possible testing outcomes: positive, negative or uncertain.**

**GRADE rating:** C - Weak

**Strength of recommendation**: This recommendation is supported with at least one study of level 3 evidence.

**Included studies:**

| **Authors and Publication Year** | **Title** | **Level of Evidence** |
| --- | --- | --- |
| Crook et al 2019 | The C9orf72 hexanucleotide repeat expansion presents a challenge for testing laboratories and genetic counseling. | 3 |
| Crook et al., 2022 | Patient and Relative Experiences and Decision-making About  Genetic Testing and Counseling for Familial ALS and FTD | 3 |
| Roggenbuck et al, 2020 | Incidence of pathogenic, likely pathogenic, and uncertain ALS variants in a clinic cohort | 3 |
| Shepheard et al, 2021 | Value of systematic genetic screening of patients with amyotrophic lateral sclerosis | 3 |
| Roggenbuck et al 2020 | Genetic Testing for Amyotrophic Lateral Sclerosis and Frontotemporal Dementia: Impact on Clinical Management. | 5 |
| Roggenbuck et al, 2017 | Genetic testing and genetic counseling for amyotrophic lateral sclerosis: an update for clinicians. | 5 |
| van der Ende et al, 2021 | Unraveling the clinical spectrum and the role of repeat length in C9ORF72 repeat expansions. | 5 |
| Volk et al, 2018 | Current knowledge and recent insights into the genetic basis of amyotrophic lateral sclerosis. | 5 |

## Recommendation 19: pALS and their families should be informed that all testing methodologies have limitations.

**GRADE rating:** C - Weak

**Strength**: This recommendation is supported with at least one study of level 3 evidence.

**Included Studies**:

| **Authors and Publication Year** | **Title** | **Level of Evidence** |
| --- | --- | --- |
| Benatar et al, 2016 | Presymptomatic ALS genetic counseling and testing | 3 |
| Crook et al 2019 | The C9orf72 hexanucleotide repeat expansion presents a challenge for testing laboratories and genetic counseling. | 3 |
| Crook et al., 2022 | Patient and Relative Experiences and Decision-making About  Genetic Testing and Counseling for Familial ALS and FTD | 3 |
| Fournier et al, 2019 | Relations between C9orf72 expansion size in blood, age at onset, age at collection and transmission across generations in patients and presymptomatic carriers | 3 |
| Jackson et al 2020 | Elevated methylation levels, reduced expression levels, and frequent contractions in a clinical cohort of C9orf72 expansion carriers. | 3 |
| Vajda et al, 2017 | Genetic testing in ALS. | 3 |
| Dharmadasa et al, 2022 | Genetic testing in motor neurone disease | 5 |
| Fong et al, 2012 | Genetic counseling for ftd/als caused by the c9orf72 hexanucleotide expansion | 5 |
| Roggenbuck et al 2020 | Genetic Testing for Amyotrophic Lateral Sclerosis and Frontotemporal Dementia: Impact on Clinical Management. | 5 |
| van der Ende et al, 2021 | Unraveling the clinical spectrum and the role of repeat length in C9ORF72 repeat expansions. | 5 |
| Veltman et al, 2013 | Challenges for implementing next-generation sequencing-based genome diagnostics: it's also the people, not just the machines. | 5 |
| Volk et al, 2018 | Current knowledge and recent insights into the genetic basis of amyotrophic lateral sclerosis. | 5 |

## Recommendation 20: All pALS who have genetic testing should receive post-test counseling.

**GRADE rating:** C - Weak

**Strength**: This recommendation is supported with at least one study of level 3 evidence.

**Included Studies**:

| **Authors and Publication Year** | **Title** | **Level of Evidence** |
| --- | --- | --- |
| Benatar et al, 2016 | Presymptomatic ALS genetic counseling and testing | 3 |
| Crook et al., 2022 | Patient and Relative Experiences and Decision-making About  Genetic Testing and Counseling for Familial ALS and FTD | 3 |
| Salman et al, 2021 | Genetic testing for amyotrophic lateral sclerosis in Canada-an assessment of current practices | 3 |
| Wagner et al, 2017 | Patients with sporadic and familial amyotrophic lateral sclerosis found value in genetic testing | 3 |
| Wagner et al, 2018 | Patients with Amyotrophic Lateral Sclerosis Have High Interest in and Limited Access to Genetic Testing | 3 |
| Anderson, 2011 | Clinical genetics of amyotrophic lateral sclerosis: What do we really know? | 5 |
| Chio el al, 2014 | Genetic counseling in ALS: facts, uncertainties and clinical suggestions. | 5 |
| Dharmadasa et al, 2022 | Genetic testing in motor neurone disease | 5 |
| Fong et al, 2012 | Genetic counseling for ftd/als caused by the c9orf72 hexanucleotide expansion | 5 |
| Hogden et al 2017 | Patient-centered decision making in amyotrophic lateral sclerosis: where are we? | 5 |
| Roggenbuck et al 2020 | Genetic Testing for Amyotrophic Lateral Sclerosis and Frontotemporal Dementia: Impact on Clinical Management. | 5 |
| Roggenbuck et al, 2017 | Genetic testing and genetic counseling for amyotrophic lateral sclerosis: an update for clinicians. | 5 |
| Volk et al, 2018 | Current knowledge and recent insights into the genetic basis of amyotrophic lateral sclerosis. | 5 |

## Recommendation 21: Post-test counseling should inform pALS with a P or LP test outcome that result typically does not allow prediction of disease course.

**GRADE rating:** B - Moderate

**Strength of recommendation**: This recommendation is supported with at least one study of level 2 evidence.

**Included Studies**:

| **Authors and Publication Year** | **Title** | **Level of Evidence** |
| --- | --- | --- |
| Rooney et al, 2017 | C9orf72 expansion differentially affects males with spinal onset amyotrophic lateral sclerosis | 2 |
| Benatar et al, 2016 | Presymptomatic ALS genetic counseling and testing | 3 |
| Crook et al 2017 | The C9orf72 hexanucleotide repeat expansion presents a challenge for testing laboratories and genetic counseling. | 3 |
| Fanos et al, 2011 | Impact of presymptomatic genetic testing for familial amyotrophic lateral sclerosis | 3 |
| Fournier et al, 2019 | Relations between C9orf72 expansion size in blood, age at onset, age at collection and transmission across generations in patients and presymptomatic carriers | 3 |
| Jackson et al 2020 | Elevated methylation levels, reduced expression levels, and frequent contractions in a clinical cohort of C9orf72 expansion carriers. | 3 |
| Ross et al, 2020 | Oligogenicity, C9orf72 expansion, and variant severity in ALS | 3 |
| Rutherford et al, 2012 | Length of normal alleles of C9ORF72 GGGGCC repeat do not influence disease phenotype | 3 |
| Vajda et al, 2017 | Genetic testing in ALS. | 3 |
| Chio el al, 2014 | Genetic counseling in ALS: facts, uncertainties and clinical suggestions. | 5 |
| Dharmadasa et al, 2022 | Genetic testing in motor neurone disease | 5 |
| Fong et al, 2012 | Genetic counseling for ftd/als caused by the c9orf72 hexanucleotide expansion | 5 |
| Roggenbuck et al 2020 | Genetic Testing for Amyotrophic Lateral Sclerosis and Frontotemporal Dementia: Impact on Clinical Management. | 5 |
| Roggenbuck et al, 2017 | Genetic testing and genetic counseling for amyotrophic lateral sclerosis: an update for clinicians. | 5 |
| Turner et al, 2017 | Genetic screening in sporadic ALS and FTD | 5 |
| van der Ende et al, 2021 | Unraveling the clinical spectrum and the role of repeat length in C9ORF72 repeat expansions. | 5 |
| Volk et al, 2018 | Current knowledge and recent insights into the genetic basis of amyotrophic lateral sclerosis. | 5 |

## Recommendation 22: Post-test counseling should inform pALS with a P or LP test outcome of the genetic risks and implications for specific family members, including the availability of pre-symptomatic testing.

**GRADE rating: C – weak**

**Strength of recommendation**: This recommendation is supported with at least one study of level 3 evidence.

**Included Studies**:

| **Authors and Publication Year** | **Title** | **Level of Evidence** |
| --- | --- | --- |
| Benatar et al, 2016 | Presymptomatic ALS genetic counseling and testing | 3 |
| Crook et al., 2022 | Patient and Relative Experiences and Decision-making About  Genetic Testing and Counseling for Familial ALS and FTD | 3 |
| Fanos et al, 2011 | Impact of presymptomatic genetic testing for familial amyotrophic lateral sclerosis | 3 |
| Jackson et al 2020 | Elevated methylation levels, reduced expression levels, and frequent contractions in a clinical cohort of C9orf72 expansion carriers. | 3 |
| Wagner et al, 2018 | Patients with Amyotrophic Lateral Sclerosis Have High Interest in and Limited Access to Genetic Testing | 3 |
| Anderson, 2011 | Clinical genetics of amyotrophic lateral sclerosis: What do we really know? | 5 |
| Dharmadasa et al, 2022 | Genetic testing in motor neurone disease | 5 |
| Fong et al, 2012 | Genetic counseling for ftd/als caused by the c9orf72 hexanucleotide expansion | 5 |
| Hogden et al 2017 | Patient-centered decision making in amyotrophic lateral sclerosis: where are we? | 5 |
| Lee et al, 2017 | Genetic testing and reproductive choice in neurological disorders. | 5 |
| Roggenbuck et al 2020 | Genetic Testing for Amyotrophic Lateral Sclerosis and Frontotemporal Dementia: Impact on Clinical Management. | 5 |
| Roggenbuck et al, 2017 | Genetic testing and genetic counseling for amyotrophic lateral sclerosis: an update for clinicians. | 5 |
| Turner et al, 2017 | Genetic screening in sporadic ALS and FTD | 5 |
| Volk et al, 2018 | Current knowledge and recent insights into the genetic basis of amyotrophic lateral sclerosis. | 5 |

**Recommendation 23: Post-test counseling should inform pALS with a P or LP test outcome of the availability of relevant observational studies, FDA approved therapies, and clinical trials.**

**GRADE rating:** C - Weak

**Strength**: This recommendation is supported with at least one study of level 3 evidence.

**Included Studies**:

| **Authors and Publication Year** | **Title** | **Level of Evidence** |
| --- | --- | --- |
| Salman et al, 2021 | Genetic testing for amyotrophic lateral sclerosis in Canada-an assessment of current practices | 3 |
| Shepheard et al, 2021 | Value of systematic genetic screening of patients with amyotrophic lateral sclerosis | 3 |
| Dharmadasa et al, 2022 | Genetic testing in motor neurone disease | 5 |
| Roggenbuck et al 2020 | Genetic Testing for Amyotrophic Lateral Sclerosis and Frontotemporal Dementia: Impact on Clinical Management. | 5 |
| Su et al, 2014 | Genetic heterogeneity of amyotrophic lateral sclerosis: Implications for clinical practice and research. | 5 |
| Turner et al, 2017 | Genetic screening in sporadic ALS and FTD | 5 |
| van der Ende et al, 2021 | Unraveling the clinical spectrum and the role of repeat length in C9ORF72 repeat expansions. | 5 |

## Recommendation 24: Post-test counseling should inform pALS with a negative outcome that the result does not exclude a genetic form of ALS

**GRADE rating:** D - Expert

**Strength**: This recommendation is supported with at least one study of level 5 evidence and expert opinion.

**Included Studies**:

| **Authors and Publication Year** | **Title** | **Level of Evidence** |
| --- | --- | --- |
| Anderson, 2011 | Clinical genetics of amyotrophic lateral sclerosis: What do we really know? | 5 |
| Chio el al, 2014 | Genetic counseling in ALS: facts, uncertainties and clinical suggestions. | 5 |
| Dharmadasa et al, 2022 | Genetic testing in motor neurone disease | 5 |
| Fong et al, 2012 | Genetic counseling for ftd/als caused by the c9orf72 hexanucleotide expansion | 5 |
| Hogden et al 2017 | Patient-centered decision making in amyotrophic lateral sclerosis: where are we? | 5 |
| Shtilbans et al, 2020 | Lifetime Risk and Heritability of Amyotrophic Lateral Sclerosis. | 5 |

## Recommendation 25: Post-test counseling should inform pALS with an uncertain result that the variant(s) may or may not be contributing to their ALS.

**GRADE rating:** C - Weak

**Strength**: This recommendation is supported with at least one study of level 3 evidence.

**Included Studies**:

| **Authors and Publication Year** | **Title** | **Level of Evidence** |
| --- | --- | --- |
| Roggenbuck et al, 2020 | Incidence of pathogenic, likely pathogenic, and uncertain ALS variants in a clinic cohort | 3 |
| Roggenbuck et al, 2021 | Amyotrophic Lateral Sclerosis Genetic Access Program: Paving the Way for Genetic Characterization of ALS in the Clinic. | 3 |
| Vajda et al, 2017 | Genetic testing in ALS. | 3 |
| Dharmadasa et al, 2022 | Genetic testing in motor neurone disease | 5 |
| Roggenbuck et al, 2020 | Genetic Testing for Amyotrophic Lateral Sclerosis and Frontotemporal Dementia: Impact on Clinical Management. | 5 |

## Recommendation 26: Post-test counseling should inform pALS that periodic reevaluation of genetic results may be appropriate, and that the interpretation of their results could change over time.

**GRADE rating:** D – Expert D - Expert

**Strength**: This recommendation is supported with at least one study of level 5 evidence and expert opinion.

**Included Studies**:

| **Authors and Publication Year** | **Title** | **Level of Evidence** |
| --- | --- | --- |
| Dharmadasa et al, 2022 | Genetic testing in motor neurone disease | 5 |

Lab Methods Recommendations

## Recommendation 27: Testing performed on DNA derived from non-CNS tissues is sufficient to establish the presence of a C9ORF72 repeat expansion.

**GRADE rating:** B- Moderate

**Strength:** This recommendation is supported by at least one study of level 2 evidence.

**Included studies**:

| **Authors and Publication Year** | **Title** | **Level of Evidence** |
| --- | --- | --- |
| Gijselinck et al., 2016 | The C9orf72 repeat size correlates with onset age of disease, DNA methylation and transcriptional downregulation of the promoter. | 2 |
| Jackson et al., 2020 | Elevated methylation levels, reduced expression levels, and frequent contractions in a clinical cohort of C9orf72 expansion carriers. | 2 |
| Fratta et al., 2015 | Screening a UK amyotrophic lateral sclerosis cohort provides evidence of multiple origins of the C9orf72 expansion. | 3 |
| Nordin et al., 2015 | Extensive size variability of the GGGGCC expansion in C9orf72 in both neuronal and non-neuronal tissues in 18 patients with ALS or FTD. | 3 |
| Pamphlett et al., 2013 | Can ALS-associated C9orf72 repeat expansions be diagnosed on a blood DNA test alone?. | 3 |
| Ross et al., 2019 | Somatic expansion of the C9orf72 hexanucleotide repeat does not occur in ALS spinal cord tissues | 3 |
| Suh et al., 2015 | Semi-automated quantification of C9orf72 expansion size reveals inverse correlation between hexanucleotide repeat number and disease duration in frontotemporal degeneration. | 3 |

## Recommendation 28: C9ORF72 testing should use a method with high sensitivity and specificity for expanded alleles.

**GRADE rating:** A- Strong

**Strength:** This recommendation is supported by one study of level 1 evidence.

**Included studies**:

| **Authors and Publication Year** | **Title** | **Level of Evidence** |
| --- | --- | --- |
| Akimoto et al., 2014 | A blinded international study on the reliability of genetic testing for GGGGCC-repeat expansions in C9orf72 reveals marked differences in results among 14 laboratories. | 1 |

**Recommendation 28a: Southern blot is an acceptable method for detecting expanded C9ORF72 alleles with high sensitivity and specificity.**

**GRADE rating:** A- Strong

**Strength:** This recommendation is supported by one study of level 1 evidence.

**Included Studies:**

| **Authors and Publication Year** | **Title** | **Level of Evidence** |
| --- | --- | --- |
| Akimoto et al., 2014 | A blinded international study on the reliability of genetic testing for GGGGCC-repeat expansions in C9orf72 reveals marked differences in results among 14 laboratories. | 1 |
| Beck et al., 2013 | Large C9orf72 hexanucleotide repeat expansions are seen in multiple neurodegenerative syndromes and are more frequent than expected in the UK population | 2 |
| Buchman et al., 2013 | Simultaneous and independent detection of C9ORF72 alleles with low and high number of GGGGCC repeats using an optimised protocol of Southern blot hybridisation. | 2 |
| Dols-Icardo et al., 2014 | Characterization of the repeat expansion size in C9orf72 in amyotrophic lateral sclerosis and frontotemporal dementia. | 2 |
| Hübers et al., 2014 | Polymerase chain reaction and Southern blot-based analysis of the C9orf72 hexanucleotide repeat in different motor neuron diseases. | 2 |
| Jackson et al., 2020 | Elevated methylation levels, reduced expression levels, and frequent contractions in a clinical cohort of C9orf72 expansion carriers. | 2 |
| Fournier et al., 2019 | Relations between C9orf72 expansion size in blood, age at onset, age at collection and transmission across generations in patients and presymptomatic carriers | 3 |
| Nordin et al., 2015 | Extensive size variability of the GGGGCC expansion in C9orf72 in both neuronal and non-neuronal tissues in 18 patients with ALS or FTD. | 3 |
| Pamphlett et al., 2013 | Can ALS-associated C9orf72 repeat expansions be diagnosed on a blood DNA test alone? | 3 |
| Suh et al., 2015 | Semi-automated quantification of C9orf72 expansion size reveals inverse correlation between hexanucleotide repeat number and disease duration in frontotemporal degeneration. | 3 |

**Recommendation 28b: Repeat-primed PCR, performed bi-directionally in some circumstances, is an acceptable method for detecting expanded C9ORF72 alleles with high sensitivity and specificity.**

**GRADE rating:** A- Strong

**Strength:** This recommendation is supported by one study of level 1 evidence.

**Included Studies**:

| **Authors and Publication Year** | **Title** | **Level of Evidence** |
| --- | --- | --- |
| Akimoto et al., 2014 | A blinded international study on the reliability of genetic testing for GGGGCC-repeat expansions in C9orf72 reveals marked differences in results among 14 laboratories. | 1 |
| Beck et al., 2013 | Large C9orf72 hexanucleotide repeat expansions are seen in multiple neurodegenerative syndromes and are more frequent than expected in the UK population | 2 |
| Buchman et al., 2013 | Simultaneous and independent detection of C9ORF72 alleles with low and high number of GGGGCC repeats using an optimised protocol of Southern blot hybridisation. | 2 |
| Cleary et al., 2016 | Improved PCR based methods for detecting C9orf72 hexanucleotide repeat expansions. | 2 |
| Corrado et al., 2018 | Characterization of the c9orf72 GC-rich low complexity sequence in two cohorts of Italian and Turkish ALS cases | 2 |
| Dols-Icardo et al., 2014 | Characterization of the repeat expansion size in C9orf72 in amyotrophic lateral sclerosis and frontotemporal dementia. | 2 |
| Hübers et al., 2014 | Polymerase chain reaction and Southern blot-based analysis of the C9orf72 hexanucleotide repeat in different motor neuron diseases. | 2 |
| Jackson et al., 2020 | Elevated methylation levels, reduced expression levels, and frequent contractions in a clinical cohort of C9orf72 expansion carriers. | 2 |
| Nordin et al., 2017 | Sequence variations in C9orf72 downstream of the hexanucleotide repeat region and its effect on repeat-primed PCR interpretation: a large multinational screening study | 2 |
| Fournier et al., 2019 | Relations between C9orf72 expansion size in blood, age at onset, age at collection and transmission across generations in patients and presymptomatic carriers | 3 |
| Nordin et al., 2015 | Extensive size variability of the GGGGCC expansion in C9orf72 in both neuronal and non-neuronal tissues in 18 patients with ALS or FTD. | 3 |
| Pamphlett et al., 2013 | Can ALS-associated C9orf72 repeat expansions be diagnosed on a blood DNA test alone? | 3 |
| Suh et al., 2015 | Semi-automated quantification of C9orf72 expansion size reveals inverse correlation between hexanucleotide repeat number and disease duration in frontotemporal degeneration. | 3 |

**Recommendation 28c: Dual-mode PCR is an acceptable method for detecting expanded C9ORF72 alleles with high sensitivity and specificity.**

**GRADE rating:** B- Moderate

**Strength:** This recommendation is supported by one study of level 2 evidence.

**Included Studies**:

| **Authors and Publication Year** | **Title** | **Level of Evidence** |
| --- | --- | --- |
| Bram et al., 2019 | Comprehensive genotyping of the C9orf72 hexanucleotide repeat region in 2095 ALS samples from the NINDS collection using a two-mode, long-read PCR assay. | 2 |

**Recommendation 28d: Expansion Hunter analysis of PCR-free whole genome sequencing data is an acceptable method for detecting expanded C9ORF72 alleles with high sensitivity and specificity.**

**GRADE rating:** B- Moderate

**Strength:** This recommendation is supported by one study of level 2 evidence.

**Included Studies**:

| **Authors and Publication Year** | **Title** | **Level of Evidence** |
| --- | --- | --- |
| Dolzhenko et al., 2017 | Detection of long repeat expansions from PCR-free whole-genome sequence data. | 2 |

## Recommendation 29: C9ORF72 testing should use a method that accurately sizes normal range alleles.

**GRADE rating:** A- Strong

**Strength:** This recommendation is supported by one study with level 1 evidence. **Included Studies**:

| **Authors and Publication Year** | **Title** | **Level of Evidence** |
| --- | --- | --- |
| Akimoto et al., 2014 | A blinded international study on the reliability of genetic testing for GGGGCC-repeat expansions in C9orf72 reveals marked differences in results among 14 laboratories. | 1 |

**Recommendation 29a: Fragment size analysis of a PCR that spans the C9ORF72 repeat, either as a stand-alone assay or part of a dual-mode PCR, is an acceptable method for sizing normal range alleles.**

**GRADE rating:** A- Strong

**Strength:** This recommendation is supported by one study with level 1 evidence**.**

**Included Studies**:

| **Authors and Publication Year** | **Title** | **Level of Evidence** |
| --- | --- | --- |
| Akimoto et al., 2014 | A blinded international study on the reliability of genetic testing for GGGGCC-repeat expansions in C9orf72 reveals marked differences in results among 14 laboratories. | 1 |
| Beck et al., 2013 | Large C9orf72 hexanucleotide repeat expansions are seen in multiple neurodegenerative syndromes and are more frequent than expected in the UK population | 2 |
| Bram et al., 2019 | Comprehensive genotyping of the C9orf72 hexanucleotide repeat region in 2095 ALS samples from the NINDS collection using a two-mode, long-read PCR assay. | 2 |
| Buchman et al., 2013 | Simultaneous and independent detection of C9ORF72 alleles with low and high number of GGGGCC repeats using an optimised protocol of Southern blot hybridisation. | 2 |
| Corrado et al., 2018 | Characterization of the c9orf72 GC-rich low complexity sequence in two cohorts of Italian and Turkish ALS cases | 2 |
| Hübers et al., 2014 | Polymerase chain reaction and Southern blot-based analysis of the C9orf72 hexanucleotide repeat in different motor neuron diseases. | 2 |
| Jackson et al., 2020 | Elevated methylation levels, reduced expression levels, and frequent contractions in a clinical cohort of C9orf72 expansion carriers. | 2 |
| Nordin et al., 2017 | Sequence variations in C9orf72 downstream of the hexanucleotide repeat region and its effect on repeat-primed PCR interpretation: a large multinational screening study | 2 |
| Nordin et al., 2015 | Extensive size variability of the GGGGCC expansion in C9orf72 in both neuronal and non-neuronal tissues in 18 patients with ALS or FTD. | 3 |

**Recommendation 29b: Expansion Hunter analysis of PCR-free whole genome sequencing data alone is an acceptable method for sizing non-expanded C9ORF72 alleles.**

**GRADE rating:** B- Moderate

**Strength:** This recommendation is supported by one study with level 2 evidence**.**

**Included Studies**:

| **Authors and Publication Year** | **Title** | **Level of Evidence** |
| --- | --- | --- |
| Dolzhenko et al., 2017 | Detection of long repeat expansions from PCR-free whole-genome sequence data. | 2 |

## Recommendation 30: Testing reports for C9ORF72 should specify the sizes of the non-expanded alleles.

**GRADE rating:** A- Strong

**Strength:** This recommendation is supported by at least 1 study with level 1 evidence.

**Included studies**:

| **Authors and Publication Year** | **Title** | **Level of Evidence** |
| --- | --- | --- |
| Akimoto et al., 2014 | A blinded international study on the reliability of genetic testing for GGGGCC-repeat expansions in C9orf72 reveals marked differences in results among 14 laboratories. | 1 |
| Iacoangeli et al., 2019 | C9orf72 intermediate expansions of 24-30 repeats are associated with ALS | 1 |
| Kaivola et al., 2019 | C9orf72 hexanucleotide repeat length in older population: normal variation and effects on cognition. | 2 |
| Crook et al., 2019 | The C9orf72 hexanucleotide repeat expansion presents a challenge for testing laboratories and genetic counseling. | 3 |
| Dedeene et al., 2019 | An ALS case with 38 (G4C2)-repeats in the C9orf72 gene shows TDP-43 and sparse dipeptide repeat protein pathology. | 3 |
| Klepek et al., 2019 | Variable reporting of C9orf72 and a high rate of uncertain results in ALS genetic testing | 3 |
| Byrne et al., 2014 | Intermediate repeat expansion length in C9orf72 may be pathological in amyotrophic lateral sclerosis | 4 |

## Recommendation 31: Labs that classify alleles as “intermediate” or “uncertain” should include a statement outlining up-to-date data regarding uncertainty of pathogenicity of these allele sizes.

**GRADE rating:** A- Strong

**Strength:**  This recommendation is supported by at least 1 study with level 1 evidence.

| **Authors and Publication Year** | **Title** | **Level of Evidence** |
| --- | --- | --- |
| Iacoangeli et al., 2019 | C9orf72 intermediate expansions of 24-30 repeats are associated with ALS | 1 |
| Kaivola et al., 2019 | C9orf72 hexanucleotide repeat length in older population: normal variation and effects on cognition. | 2 |
| Crook et al., 2019 | The C9orf72 hexanucleotide repeat expansion presents a challenge for testing laboratories and genetic counseling. | 3 |
| Dedeene et al., 2019 | An ALS case with 38 (G4C2)-repeats in the C9orf72 gene shows TDP-43 and sparse dipeptide repeat protein pathology. | 3 |
| Klepek et al., 2019 | Variable reporting of C9orf72 and a high rate of uncertain results in ALS genetic testing | 3 |
| Byrne et al., 2014 | Intermediate repeat expansion length in C9orf72 may be pathological in amyotrophic lateral sclerosis | 4 |

## Recommendation 32: Labs reporting C9ORF72 repeat expansions should include a statement clearly outlining the maximum number of repeats detectable by the assay employed (e.g. >55 repeats; >145 repeats; 1500-2500 depending on the method).

**GRADE rating:** D- Expert

**Strength**: There are no studies addressing this recommendation.

## Recommendation 33: The interrogation of non-C9ORF72 ALS genes should utilize simultaneous sequencing methods (e.g. panel, exome, genome) rather than sequential gene sequencing.

**GRADE rating:** B - Moderate

**Strength**: This recommendation is supported by at least one study with level 2 evidence.

**Included studies**:

| **Authors and Publication Year** | **Title** | **Level of Evidence** |
| --- | --- | --- |
| Dekker et al., 2016 | Large-scale screening in sporadic amyotrophic lateral sclerosis identifies genetic modifiers in C9orf72 repeat carriers. | 2 |
| Morgan et al., 2017 | A comprehensive analysis of rare genetic variation in amyotrophic lateral sclerosis in the UK. | 2 |
| Nakamura et al., 2016 | Next-generation sequencing of 28 ALS-related genes in a Japanese ALS cohort. | 2 |
| Pecoraro et al., 2020 | The NGS technology for the identification of genes associated with the ALS. A systematic review | 2 |
| Kenna et al., 2013 | Delineating the genetic heterogeneity of ALS using targeted high-throughput sequencing | 3 |
| Keogh et al., 2018 | Oligogenic genetic variation of neurodegenerative disease genes in 980 postmortem human brains. | 3 |
| Lamp et al., 2018 | Twenty years of molecular analyses in amyotrophic lateral sclerosis: genetic landscape of Italian patients | 3 |
| Lattante et al., 2020 | High-Throughput Genetic Testing in ALS: The Challenging Path of Variant Classification Considering the ACMG Guidelines. | 3 |
| Ross et al., 2020 | Oligogenicity, C9orf72 expansion, and variant severity in ALS | 3 |
| van Blitterswijk et al., 2012 | Evidence for an oligogenic basis of amyotrophic lateral sclerosis. | 3 |
| Bury et al., 2016 | Oligogenic inheritance of optineurin (OPTN) and C9ORF72 mutations in ALS highlights localisation of OPTN in the TDP-43-negative inclusions of C9ORF72-ALS. | 4 |
| Cady et al., 2015 | Amyotrophic lateral sclerosis onset is influenced by the burden of rare variants in known amyotrophic lateral sclerosis genes. | 4 |
| Cooper-Knock et al., 2017 | Targeted Genetic Screen in Amyotrophic Lateral Sclerosis Reveals Novel Genetic Variants with Synergistic Effect on Clinical Phenotype. | 4 |
| Couthouis et al., 2014 | Targeted exon capture and sequencing in sporadic amyotrophic lateral sclerosis. | 4 |
| Gibson et al., 2017 | The evolving genetic risk for sporadic ALS | 4 |
| Krüger et al., 2016 | Rare Variants in Neurodegeneration Associated Genes Revealed by Targeted Panel Sequencing in a German ALS Cohort. | 4 |
| Lattante et al., 2019 | Coexistence of variants in TBK1 and in other ALS-related genes elucidates an oligogenic model of pathogenesis in sporadic ALS | 4 |
| Morgan et al., 2015 | Investigation of next-generation sequencing technologies as a diagnostic tool for amyotrophic lateral sclerosis | 4 |
| Naruse et al., 2019 | Burden of rare variants in causative genes for amyotrophic lateral sclerosis (ALS) accelerates age at onset of ALS | 4 |
| Pang et al., 2017 | Burden of rare variants in ALS genes influences survival in familial and sporadic ALS. | 4 |
| Zhang et al., 2018 | Screening for possible oligogenic pathogenesis in Chinese sporadic ALS patients. | 4 |

## Recommendation 34: Based on ClinGen classifications, ALS gene panel reports should clearly differentiate between genes that are causal for ALS and those genes where the evidence is sparse, conflicting or insufficient.

**GRADE rating:** D- Expert

**Strength**: There are no studies supporting this recommendation

**Recommendation 35: When targeted-capture, whole-exome, or whole genome methods are used, targeted gene regions that were not adequately assessed should be interrogated further or highlighted in the report.**

**GRADE rating:** D- Expert

**Strength:** There are no studies supporting this recommendation.
